# Supplementary material for: Pt-doped Ru nanoparticles loaded on ‘black gold’ plasmonic nanoreactors as air stable reduction catalysts
Source: Nat Commun. 2024 Jan 24;15:713. doi: 10.1038/s41467-024-44954-4 (PMC10808126; doi:10.1038/s41467-024-44954-4)
Supplement: Supplementary file 1 — Supplementary Information [file 41467_2024_44954_MOESM1_ESM.pdf]

Supplementary Materials

Pt-Doped Ru Nanoparticles Loaded on 'Black Gold' Plasmonic Nanoreactors as Air Stable Reduction Catalysts

Gunjan Sharma,[a] Rishi Verma,[a] Shinya Masuda,[b] Khaled Mohamed Badawy,[c] Nirpendra Singh,[c] Tatsuya Tsukuda,*[b] Vivek Polshettiwar*[a]

[a]Department of Chemical Sciences, Tata Institute of Fundamental Research, Mumbai, 40005 India
[b]Department of Chemistry, Graduate School of Science, The University of Tokyo, Tokyo, 113-0033 Japan
[c]Department of Physics, Khalifa University, Abu Dhabi, 127788 United Arab Emirates

Email: vivekpol@tifr.res.in, tsukuda@chem.s.u-tokyo.ac.jp

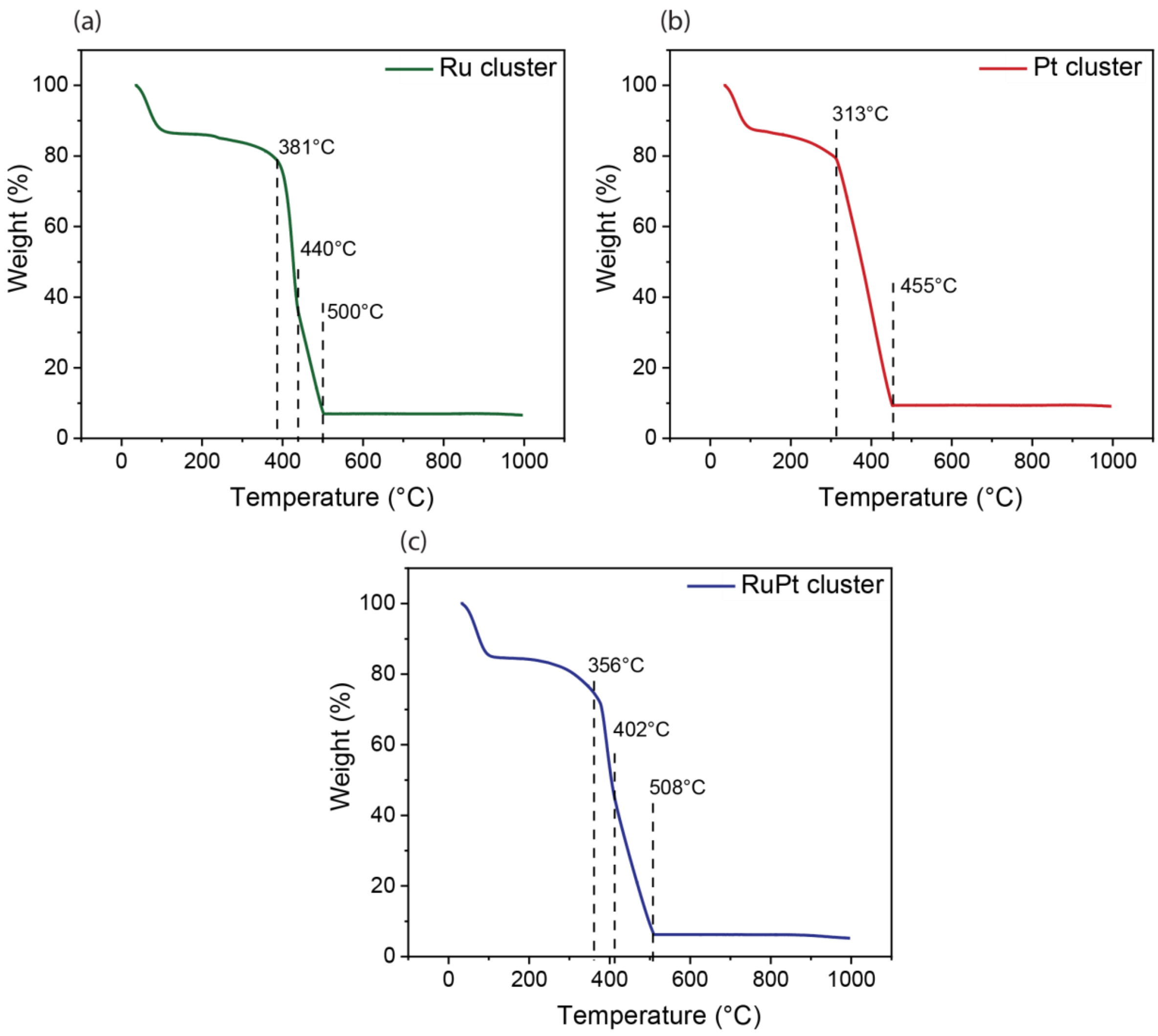

Figure S1. Thermogravimetric weight loss profile in airflow (40 mL min^{-1}) from 30 °C to 1000 °C (ramp-10 °C min^{-1}) showing the loss of capping agent, PVP for (a) Ru clusters; (b) Pt clusters and (c) RuPt bimetallic clusters.

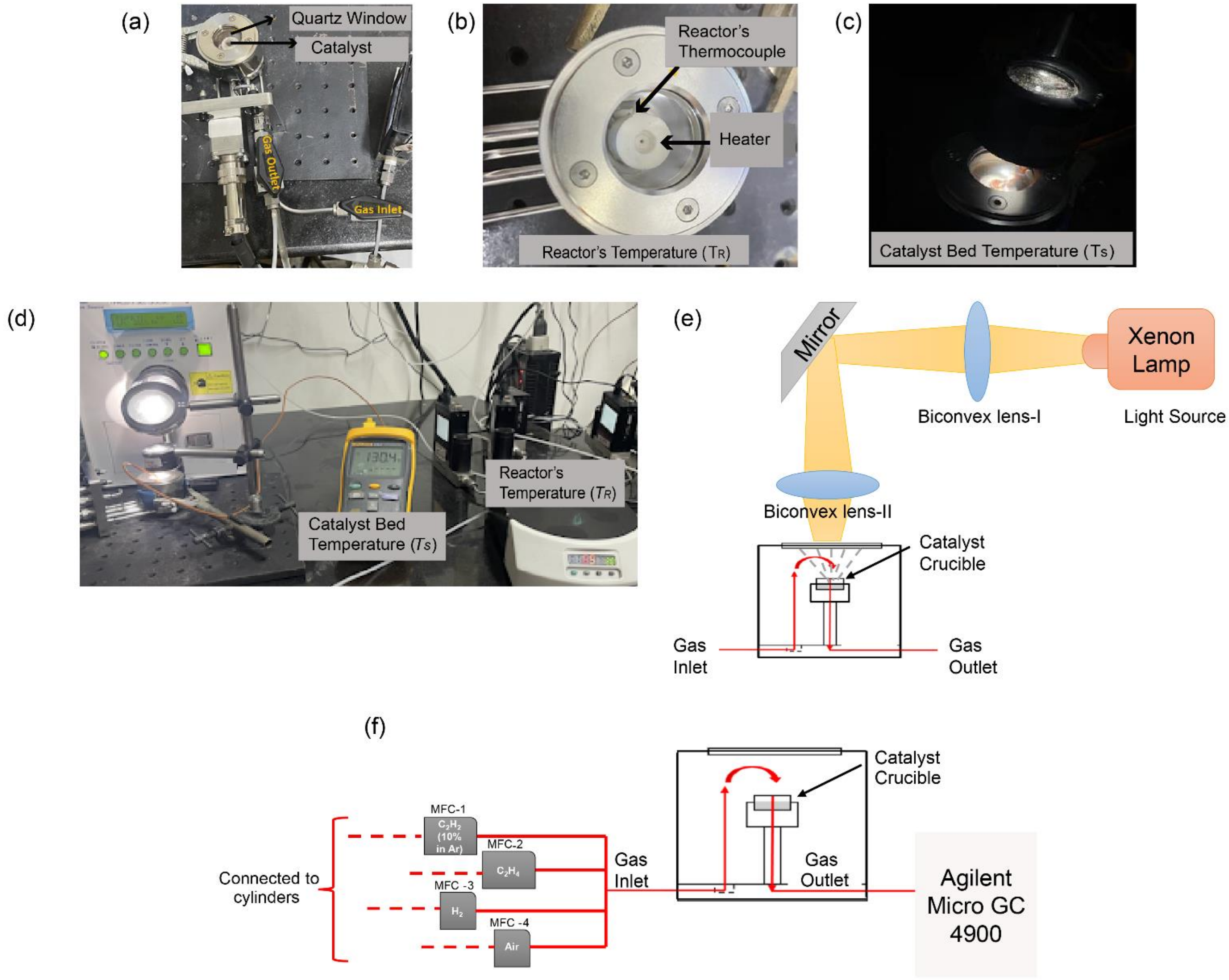

Figure S2. Photocatalytic C_2H_2 semi-hydrogenation experimental set-up. (a) photograph of pike flow reactor (top view) showing the gas inlet, outlet, quartz window, and porous Al_2O_3 crucible containing ~5 mg catalyst; (b) Zoomed-in version of the pike flow reactor chamber showing the heater and reactor's inbuilt thermocouple for temperature measurement; (c) Measurement of catalyst bed temperature (Ts) using external thermocouple under visible light illumination; (d) A typical photocatalytic reaction setup employed for *Ts* measurement showing temperature readings (using an external thermocouple, Ts) and reactor's temperature (using reactor's inbuilt thermocouple, T_R); (e) focused light path diagram enabled by the lens and mirror assembly; (f) sketch of pike reactor with gas lines and interaction of gas flow with the catalyst powder in the crucible.

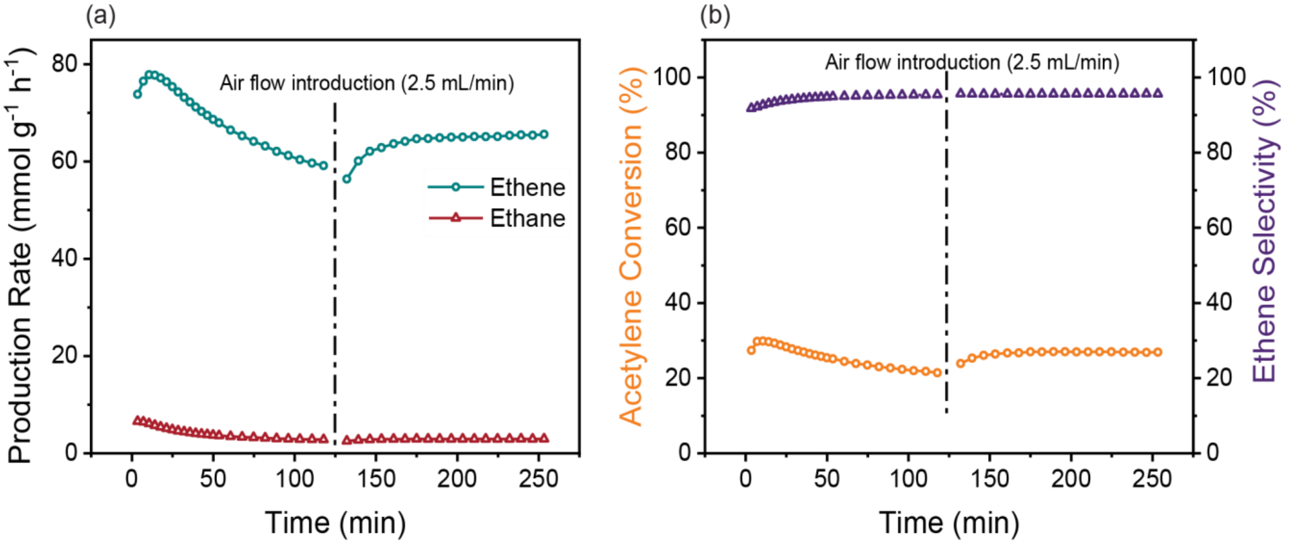

Figure S3. Acetylene semi-hydrogenation over DPC/RuPt-3-Calc (a) ethene and ethane production rate; (b) acetylene conversion and ethene selectivity, with and without airflow along with reactant gas flow of 10 mL min^{-1} C_2H_2 (10 % in Ar), 5 mL min^{-1} H_2 balanced by Ar to make the total flow 100 mL min^{-1} at 1 bar pressure and visible light illumination (400–1100 nm) with a light intensity of 2.7 W cm^{-2}.

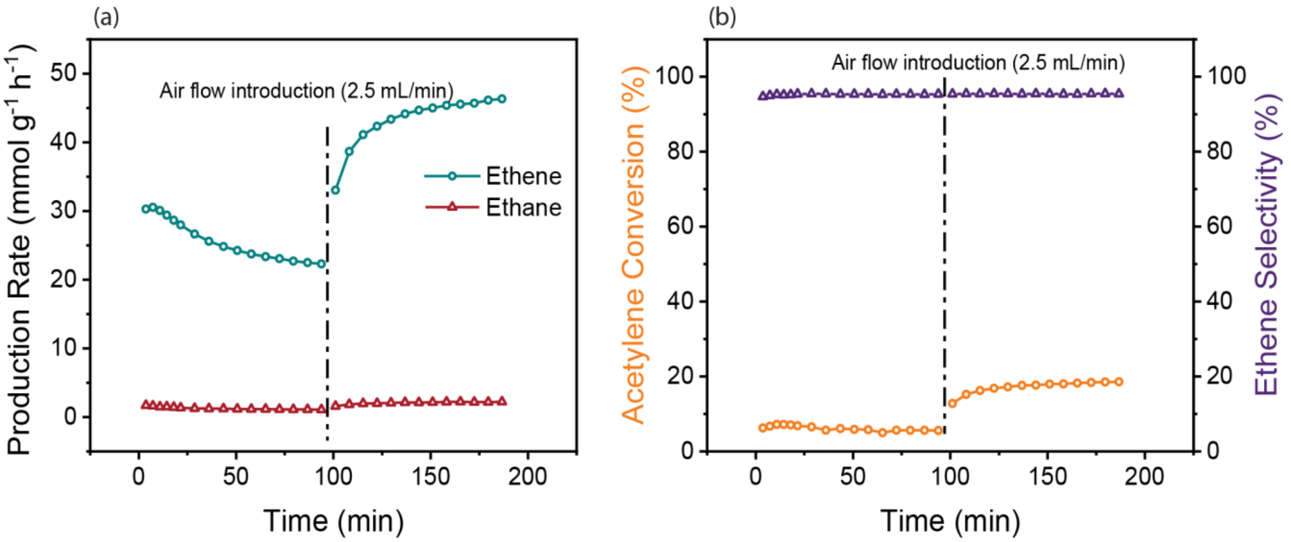

Figure S4. Acetylene semi-hydrogenation over DPC/RuPt-3-reduced (after treating DPC/RuPt-3-Calc in 50 mL min^{-1} H_2 flow at 400°C for 3 h) (a) ethene and ethane production rate; (b) acetylene conversion and ethene selectivity, with and without airflow along with reactant gas flow of 10 mL min^{-1} C_2H_2 (10 % in Ar), 5 mL min^{-1} H_2 balanced by Ar to make the total flow 100 mL min^{-1} at 1 bar pressure and visible light illumination (400–1100 nm) with a light intensity of 2.7 W cm^{-2}.

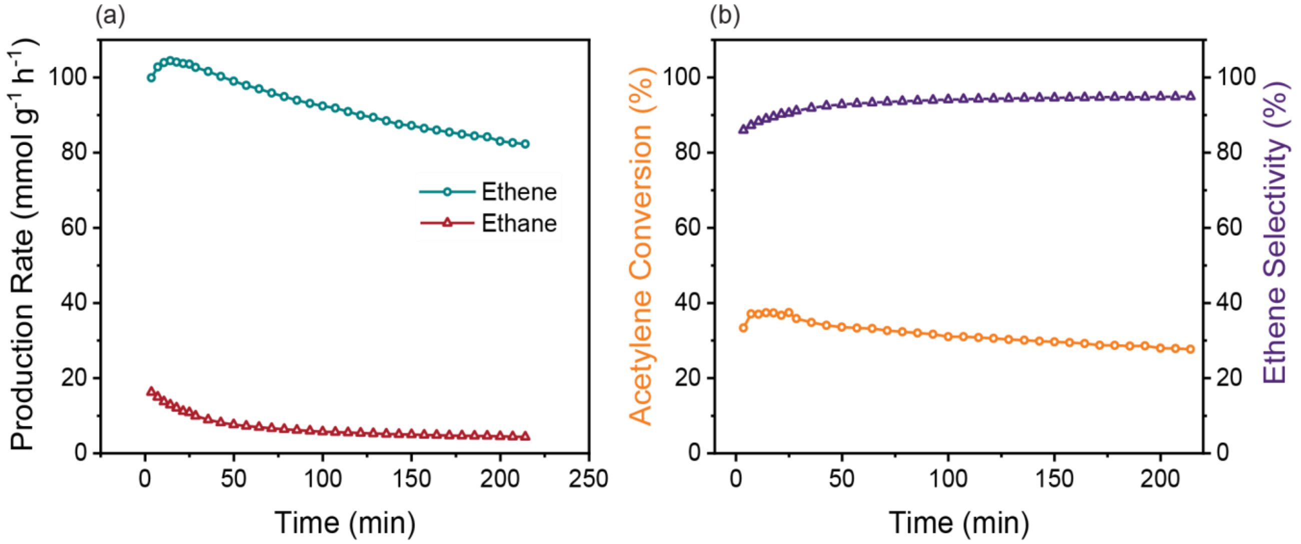

Figure S5. Acetylene semi-hydrogenation over DPC/RuPt-5-Calc (a) ethene and ethane production rate; (b) acetylene conversion and ethene selectivity, with reactant gas flow of 10 mL min^{-1} C_2H_2 (10 % in Ar), 5 mL min^{-1} H_2 balanced by Ar to make the total flow 100 mL min^{-1} at 1 bar pressure and visible light illumination (400–1100 nm) with a light intensity of 2.7 W cm^{-2}.

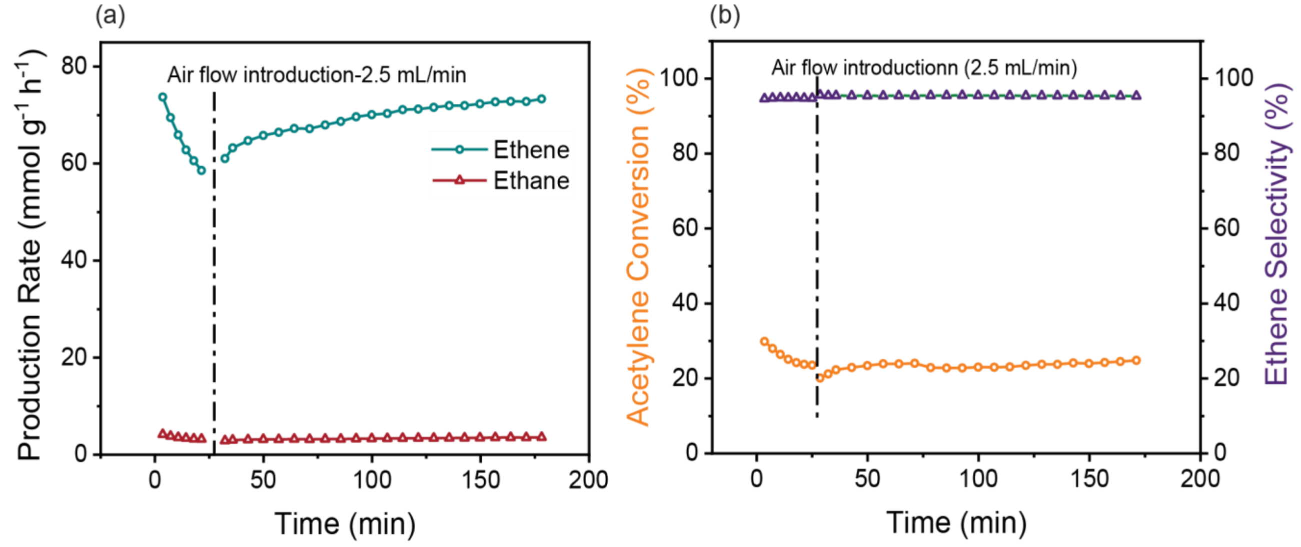

Figure S6. Acetylene semi-hydrogenation over DPC/RuPt-5-reduced (after treating DPC/RuPt-5-Calc in 50 mL min^{-1} H_2 flow at 400 °C for 3 h) (a) ethene and ethane production rate; (b) acetylene conversion and ethene selectivity, with and without airflow along with reactant gas flow of 10 mL min^{-1} C_2H_2 (10 % in Ar), 5 mL min^{-1} H_2 balanced by Ar to make the total flow 100 mL min^{-1} at 1 bar pressure and visible light illumination (400–1100 nm) with a light intensity of 2.7 W cm^{-2}.

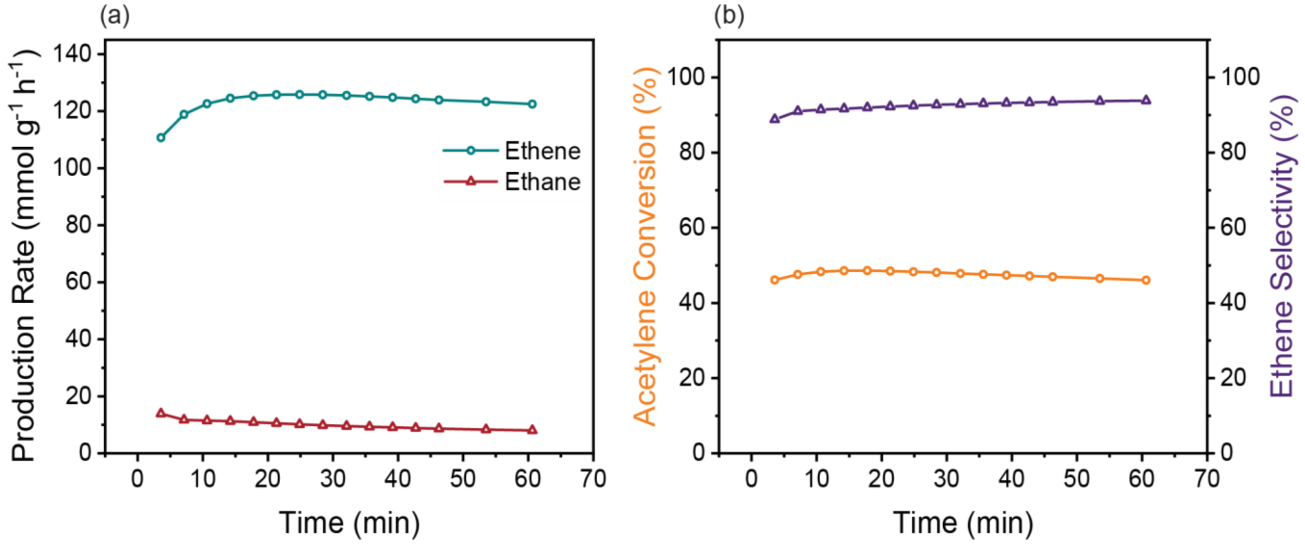

Figure S7. Acetylene semi-hydrogenation over DPC/RuPt-10-Calc (a) ethene and ethane production rate; (b) acetylene conversion and ethene selectivity, with reactant gas flow of 10 mL min^{-1} C_2H_2 (10 % in Ar), 5 mL min^{-1} H_2 balanced by Ar to make the total flow 100 mL min^{-1} at 1 bar pressure and visible light illumination (400–1100 nm) with a light intensity of 2.7 W cm^{-2}.

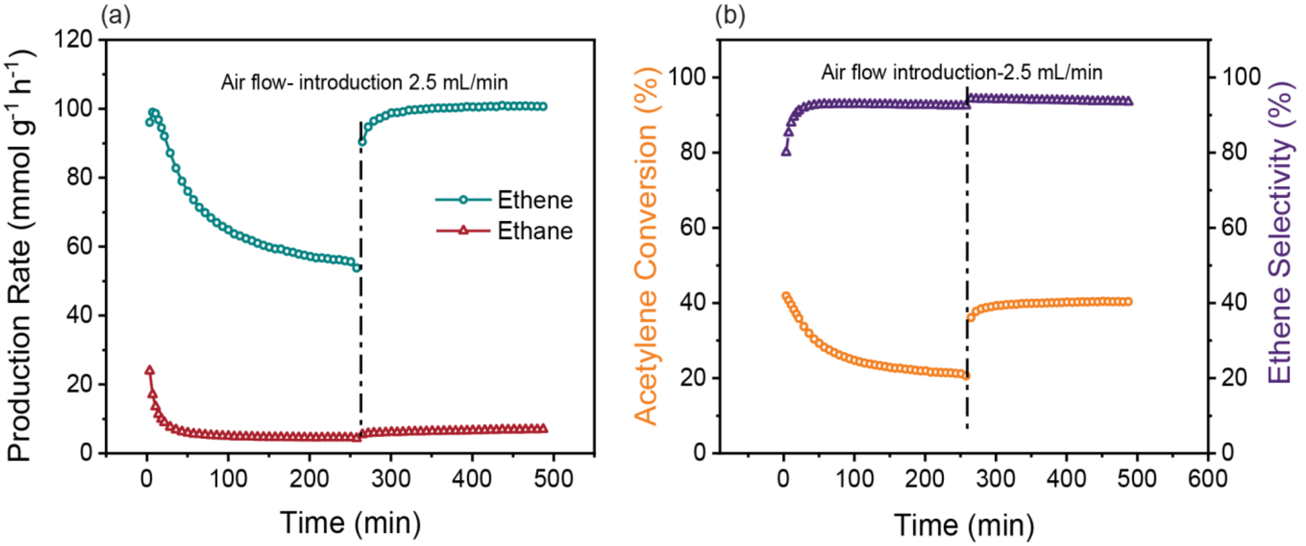

Figure S8. Acetylene semi-hydrogenation over DPC/RuPt-10-reduced (after treating DPC/RuPt-10-Calc in 50 mL min^{-1} H_2 flow at 400 °C for 3 h) (a) ethene and ethane production rate; (b) acetylene conversion and ethene selectivity, with and without airflow along with reactant gas flow of 10 mL min^{-1} C_2H_2 (10 % in Ar), 5 mL min^{-1} H_2 balanced by Ar to make the total flow 100 mL min^{-1} at 1 bar pressure and visible light illumination (400–1100 nm) with a light intensity of 2.7 W cm^{-2}.

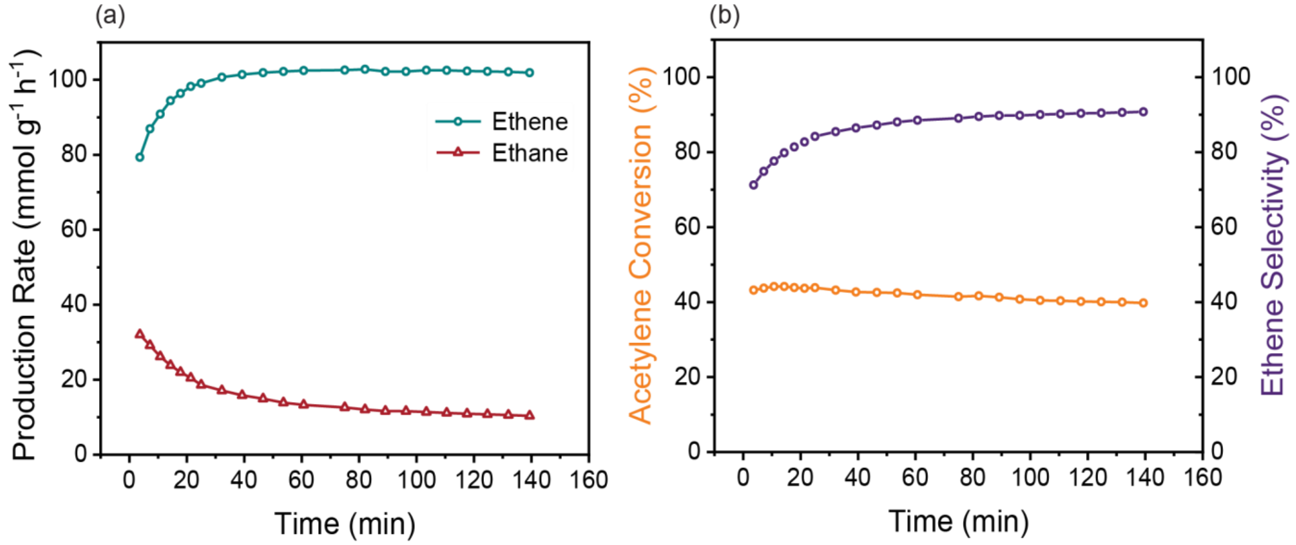

Figure S9. Acetylene semi-hydrogenation over DPC/RuPt-20-Calc (a) ethene and ethane production rate; (b) acetylene conversion and ethene selectivity, with reactant gas flow of 10 mL min^{-1} C_2H_2 (10 % in Ar), 5 mL min^{-1} H_2 balanced by Ar to make the total flow 100 mL min^{-1} at 1 bar pressure and visible light illumination (400–1100 nm) with a light intensity of 2.7 W cm^{-2}.

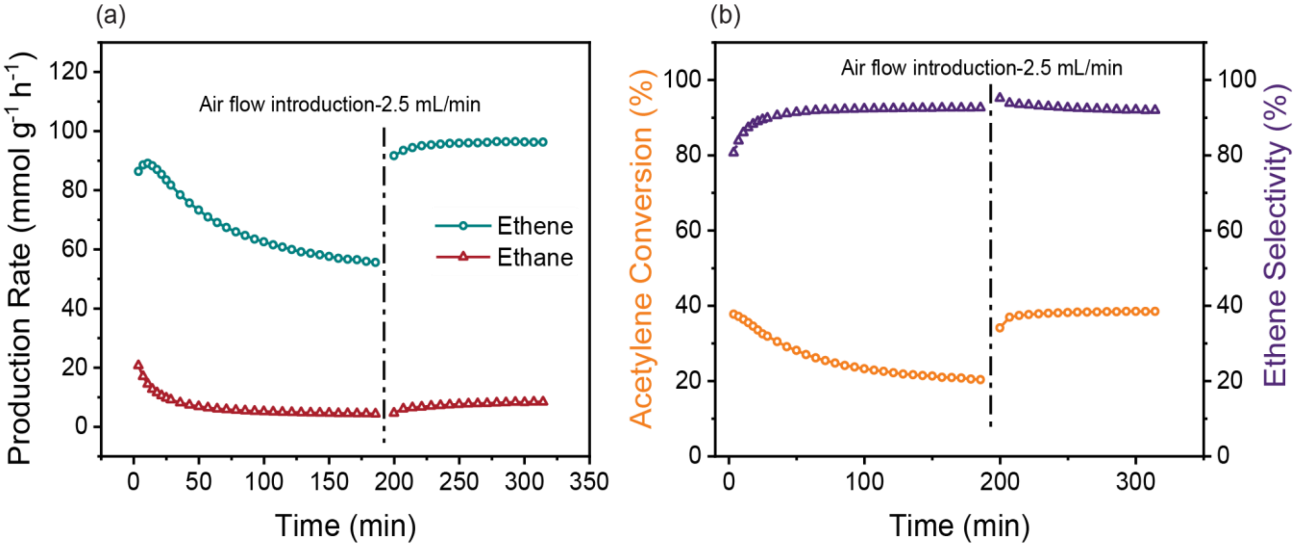

Figure S10. Acetylene semi-hydrogenation over DPC/RuPt-20-reduced (after treating DPC/RuPt-20-Calc in 50 mL min^{-1} H_2 flow at 400 °C for 3 h) (a) ethene and ethane production rate; (b) acetylene conversion and ethene selectivity, with and without airflow along with reactant gas flow of 10 mL min^{-1} C_2H_2 (10 % in Ar), 5 mL min^{-1} H_2 balanced by Ar to make the total flow 100 mL min^{-1} at 1 bar pressure and visible light illumination (400–1100 nm) with a light intensity of 2.7 W cm^{-2}.

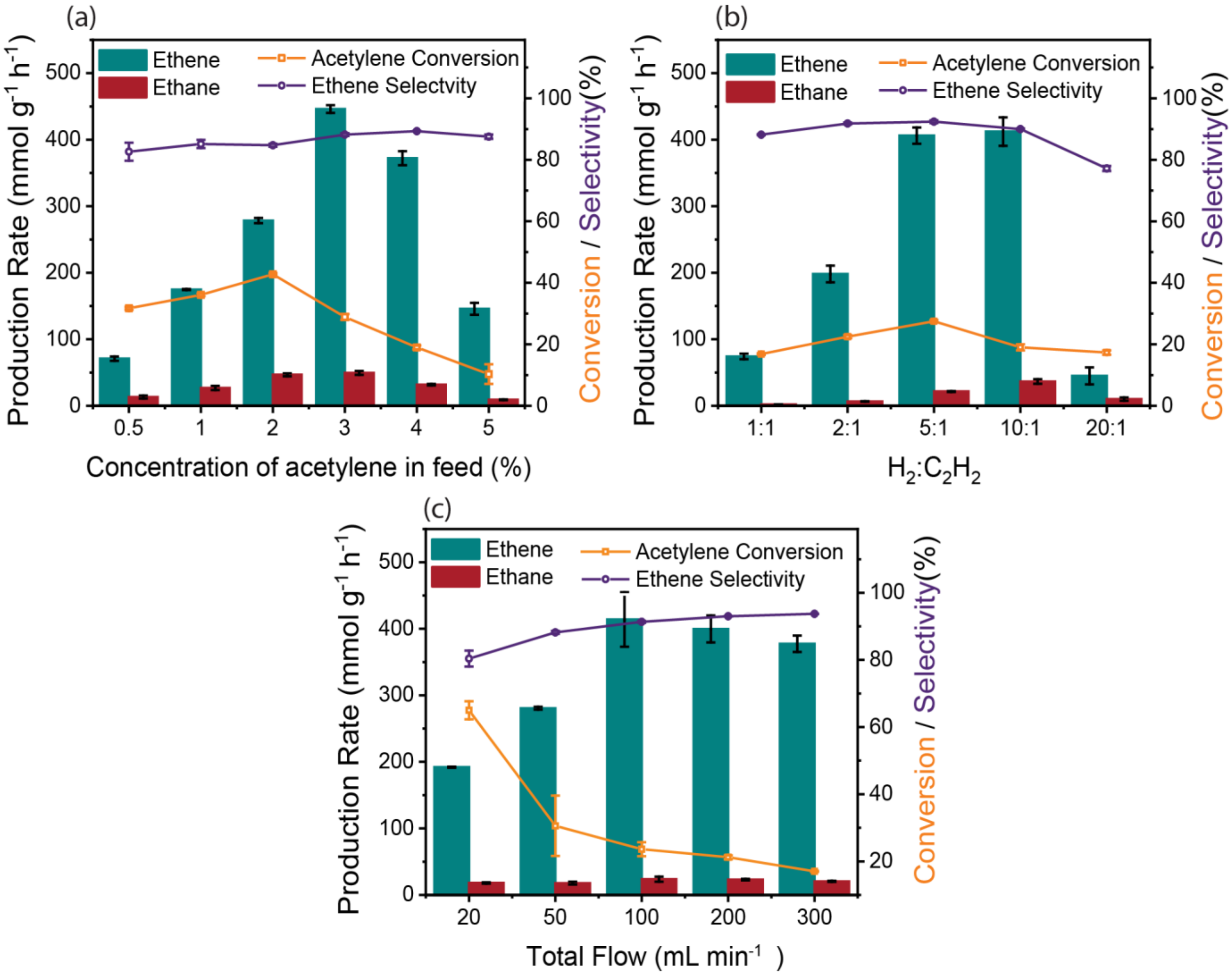

Figure S11. Acetylene semi-hydrogenation over DPC/RuPt-10-Calc (a) at different concentrations of acetylene in the total feed at a fixed H_2:C_2H_2 ratio of 5:1, the total flow of 100 mL min^{-1}, visible light illumination (400–1100 nm) with a light intensity of 2.7 W cm^{-2} at 1bar pressure; (b) at different H_2:C_2H_2 ratios with acetylene concentration in the feed was fixed to be 3 %, total flow of 100 mL min^{-1}, visible light illumination (400–1100 nm) with a light intensity of 2.7 W cm^{-2} at 1 bar pressure; (c) at different total flows with C_2H_2:H_2:Ar = 3:15:82, visible light illumination (400–1100 nm) with a light intensity of 2.7 W cm^{-2} at 1 bar pressure.

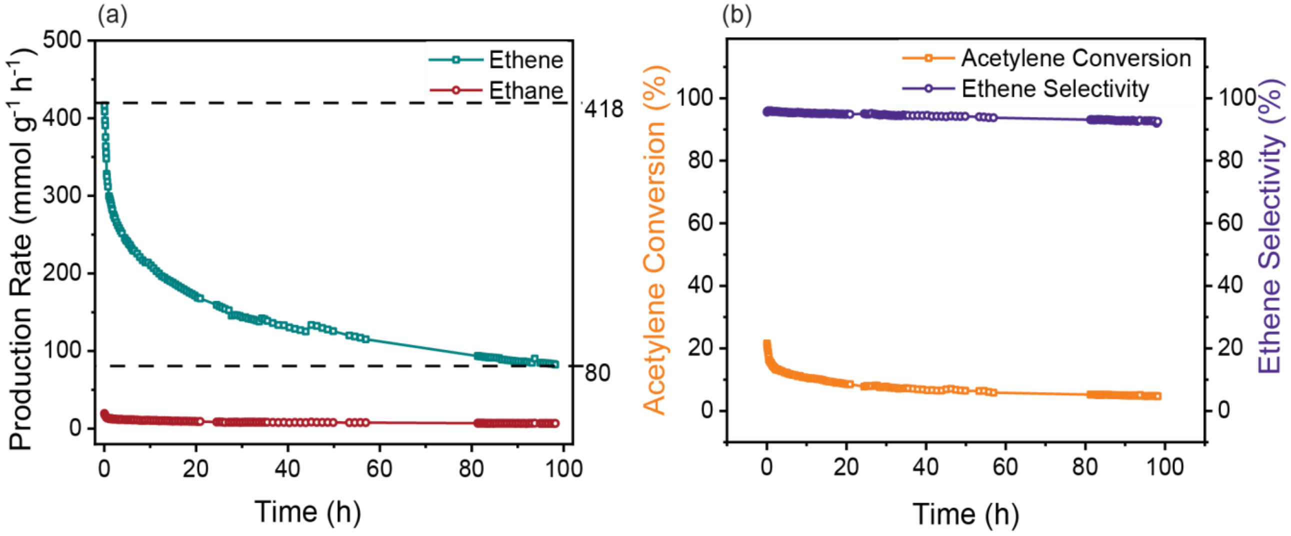

Figure S12. Long-term stability of DPC/RuPt-10-Calc catalyst, (a) ethene and ethane production rate; (b) acetylene conversion and ethene selectivity for non-competitive acetylene semi-hydrogenation with 400–1100 nm illumination at 2.7 W cm^{-2}, 1 bar pressure, optimized flow conditions- C_2H_2:H_2:Ar = 3:15:82, the total flow of 100 mL min^{-1}.

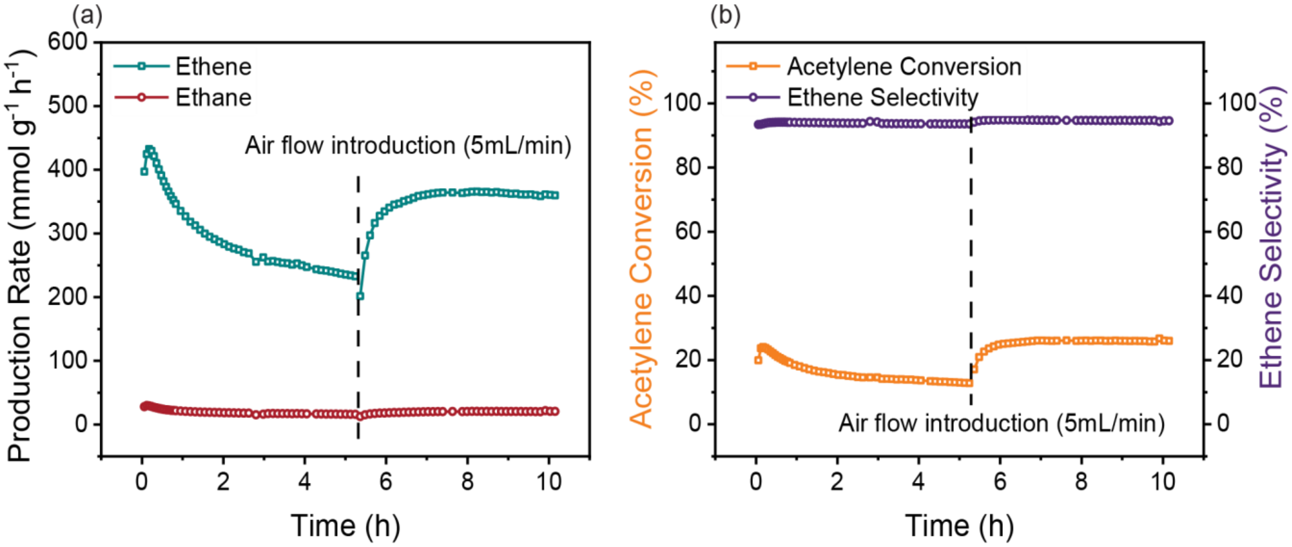

Figure S13. Acetylene semi-hydrogenation over DPC/RuPt-10-Calc (after treating deactivated DPC/RuPt-10-Calc (100 h) in 50 mL min^{-1} air flow at 400 °C for 3h (a) ethene and ethane production rate; (b) acetylene conversion and ethene selectivity, with and without airflow along with reactant gas flow of 30 mL min^{-1} C_2H_2 (10 % in Ar), 15 mL min^{-1} H_2 balanced by Ar to make the total flow 100 mL min^{-1}at 1 bar pressure and visible light illumination (400–1100nm) with a light intensity of 2.7 W cm^{-2}.

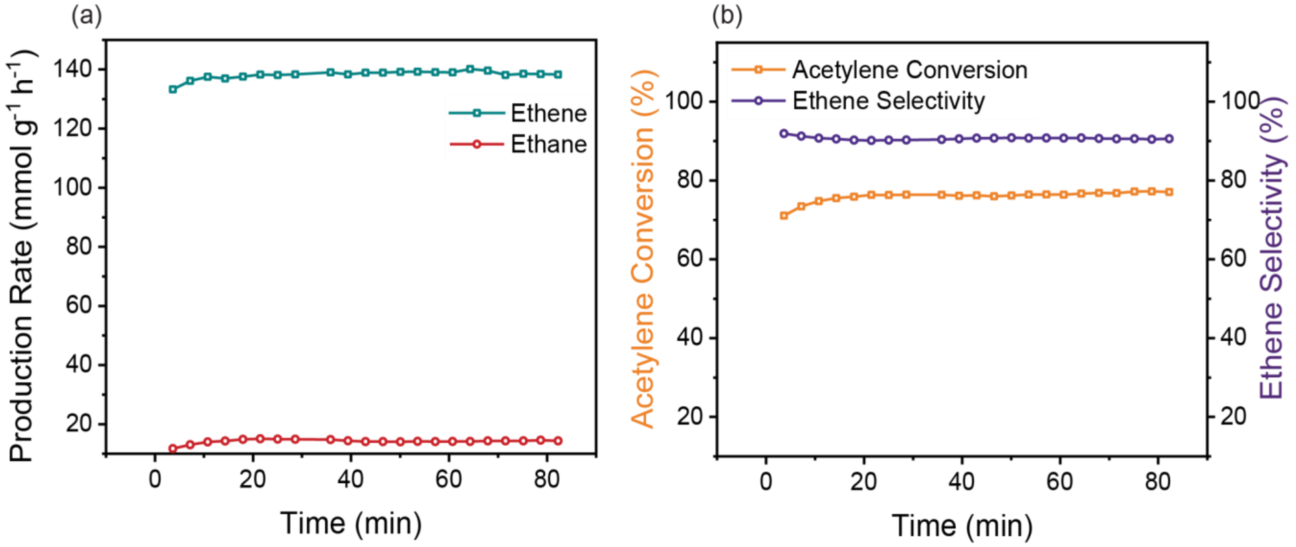

Figure S14. (a) Production rate of ethene and ethane; (b) acetylene conversion and ethene selectivity at a total flow rate of 10.5 mL min^{-1} (1.5 mL min^{-1} H_2, 3 mL min^{-1} C_2H_2 (10 % in Ar), 5.5 mL min^{-1} Ar, 0.5 mL min^{-1}air) and visible light illumination (400–1100 nm) with a light intensity of 2.7 W cm^{-2}.

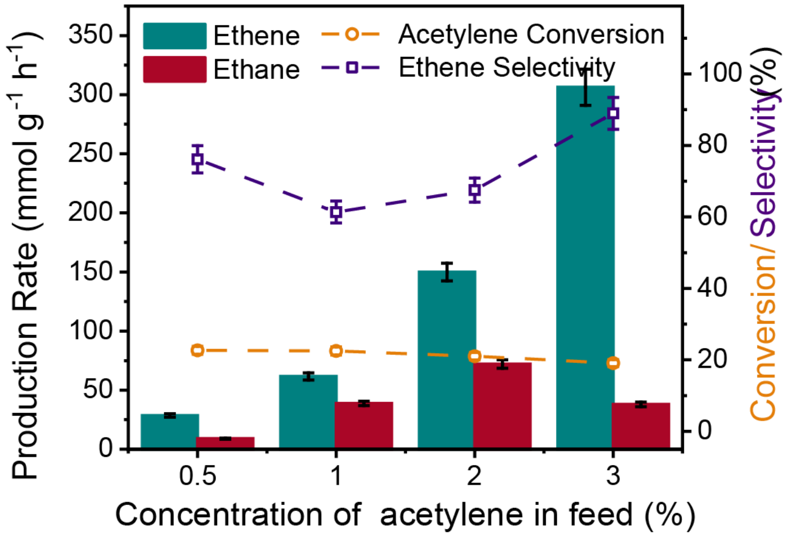

Figure S15. Production rate of ethene and ethane, acetylene conversion, and ethene selectivity for acetylene semi-hydrogenation in excess ethene over DPC/RuPt-10-Calc at different concentrations of acetylene in feed (H_2:C_2H_2=5:1, C_2H_4:C_2H_2=20:1 and 1320000 mL g^{-1} h^{-1} GHSV, visible light illumination (400–1100 nm) with light intensity of 2.7 W cm^{-2}).

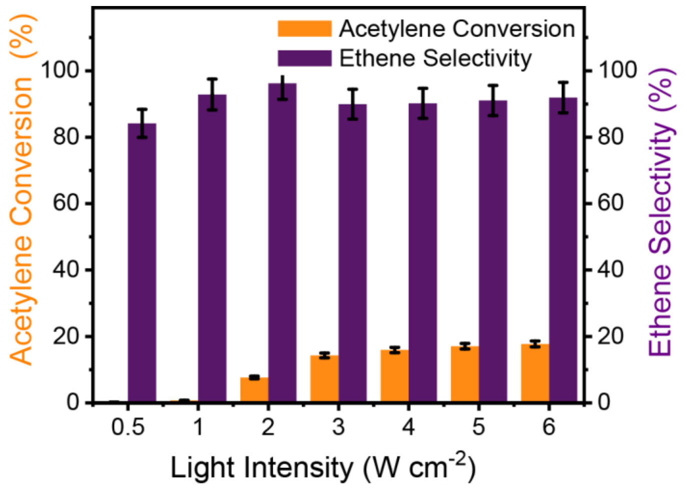

Figure S16. Acetylene conversion and ethene selectivity for acetylene semi-hydrogenation in excess ethene over DPC/RuPt-10-Calc at different light intensities (400-1100 nm) (Flow: 3 % acetylene in feed, H_2:C_2H_2=5:1, C_2H_4:C_2H_2=20:1 and 1320000 mL g^{-1} h^{-1} GHSV).

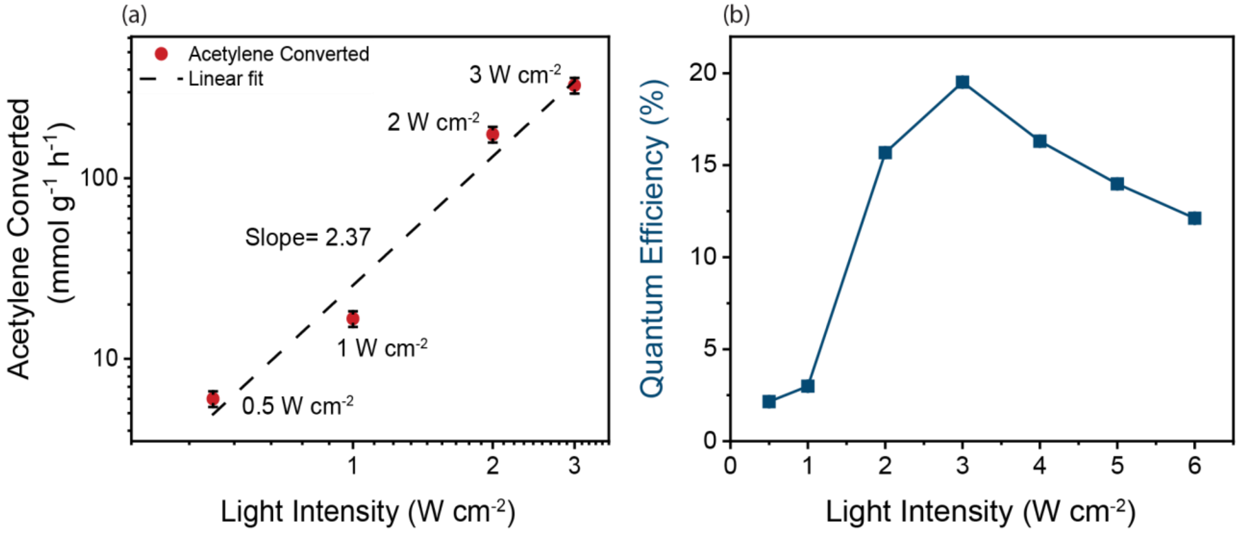

Figure S17. (a) Light intensity-activity relation (log scale) for DPC/RuPt-10-Calc; (b) Quantum efficiency at different light intensities.

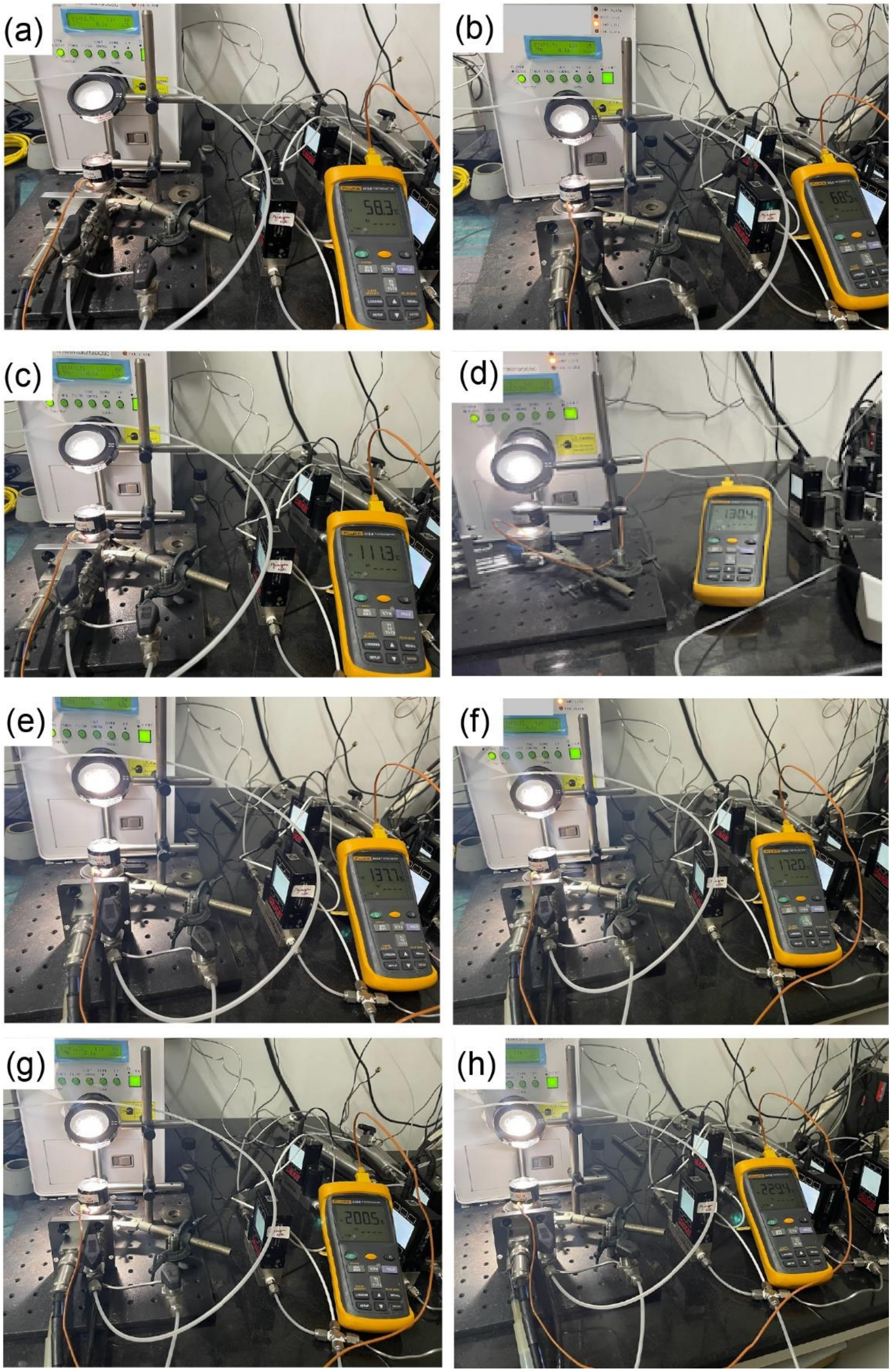

Figure S18. Measurement of surface temperatures using external thermocouple at different light intensities (in W cm^{-2}), (a) 0.5; (b) 1; (c) 2; (d) 2.7; (e) 3; (f) 4; (g) 5; (h) 6 with the optimized reactant gas flow- 30 mL min^{-1} C_2H_2 (10 % in Ar), 60 mL min^{-1} C_2H_4, 15 mL min^{-1} H_2, 5 mL min^{-1} air at 1 bar pressure.

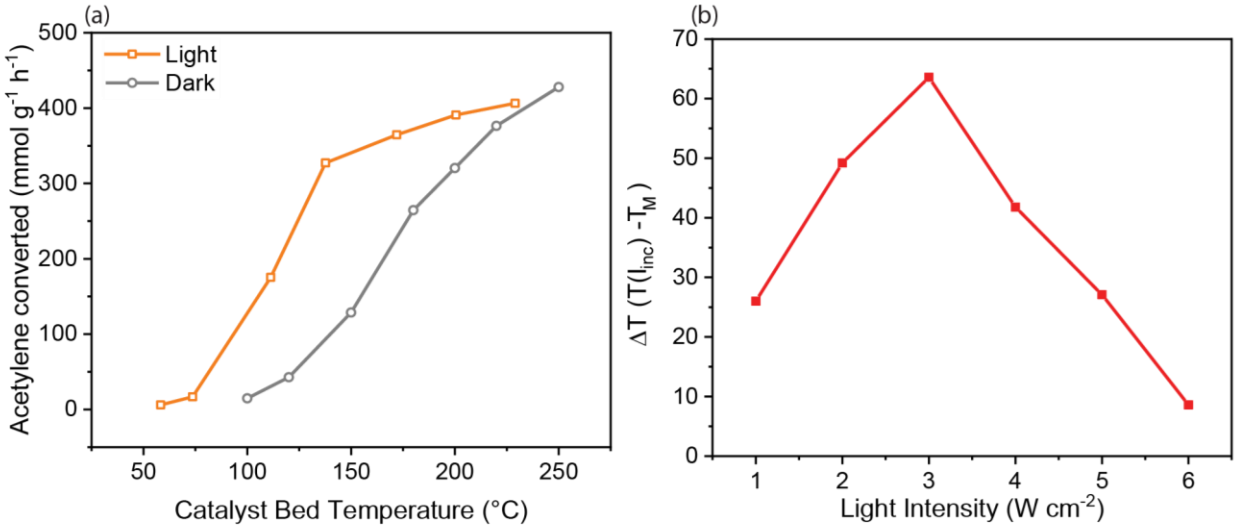

Figure S19. (a) Acetylene conversion in light and dark versus the catalyst bed temperature; (b) Temperature difference between $T(I_{inc})$ and T_M versus light intensity.

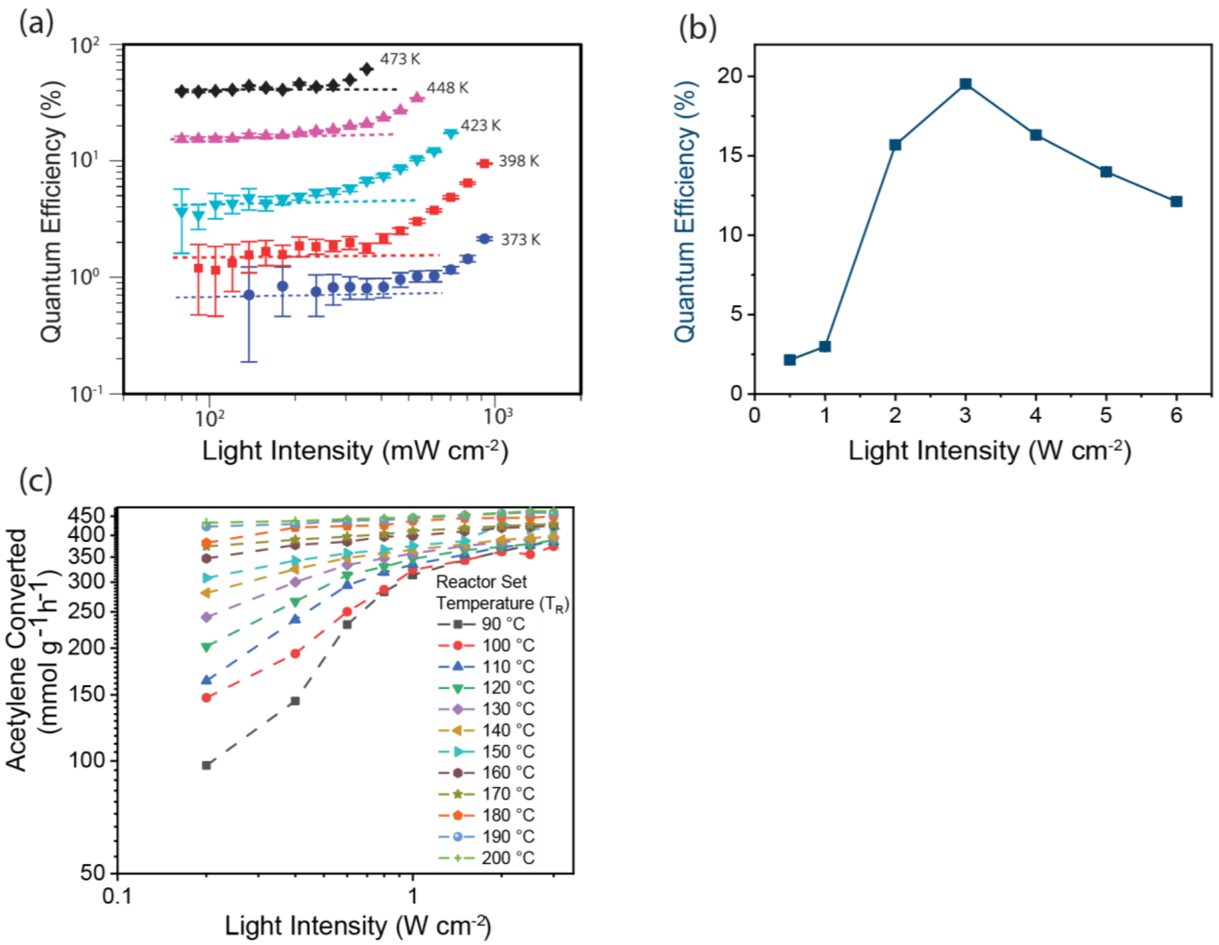

Figure S20. (a) Quantum efficiency (%) as a function of intensity for various temperatures. The dotted lines show the quantum efficiency observed at source intensities <~300 mW cm^{-2} for ethene epoxidation over Ag nanocubes (reproduced from reference-1, Copyright 2012 Springer Nature); (b) Quantum efficiency at different light intensities; (c) moles of acetylene converted (log scale) v/s light intensity (log scale) used at different reactor temperatures (T_R), for acetylene semi hydrogenation in excess ethene over DPC/RuPt-10-Calc.

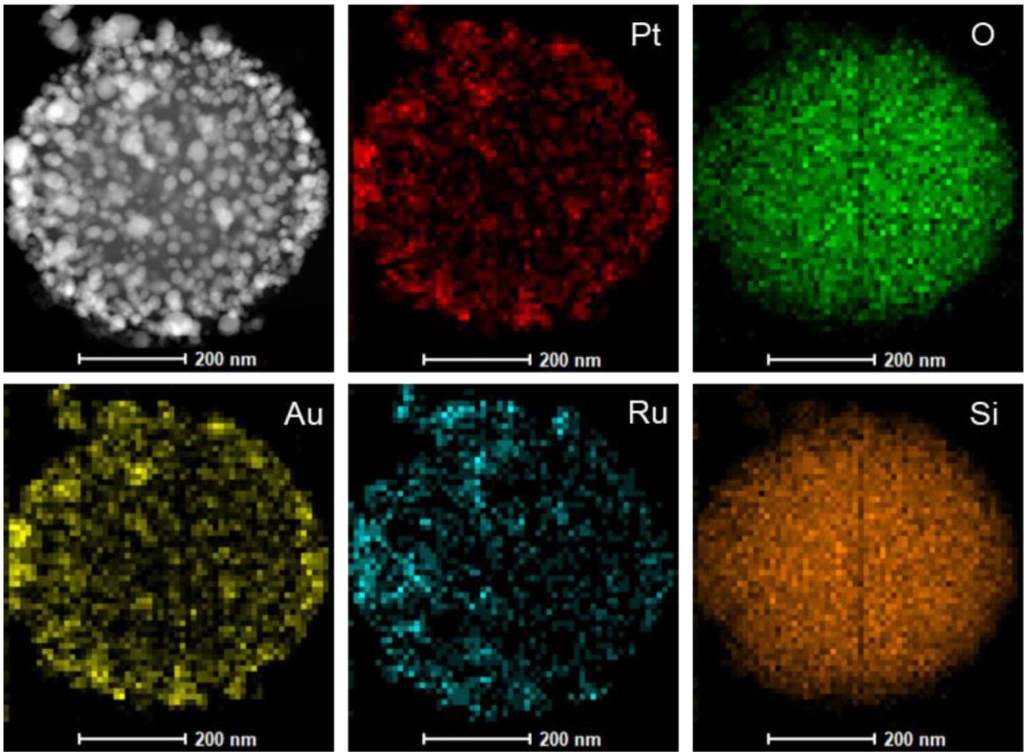

Figure S21. HAADF-STEM image and EDS elemental maps of DPC/RuPt-10 spent catalyst.

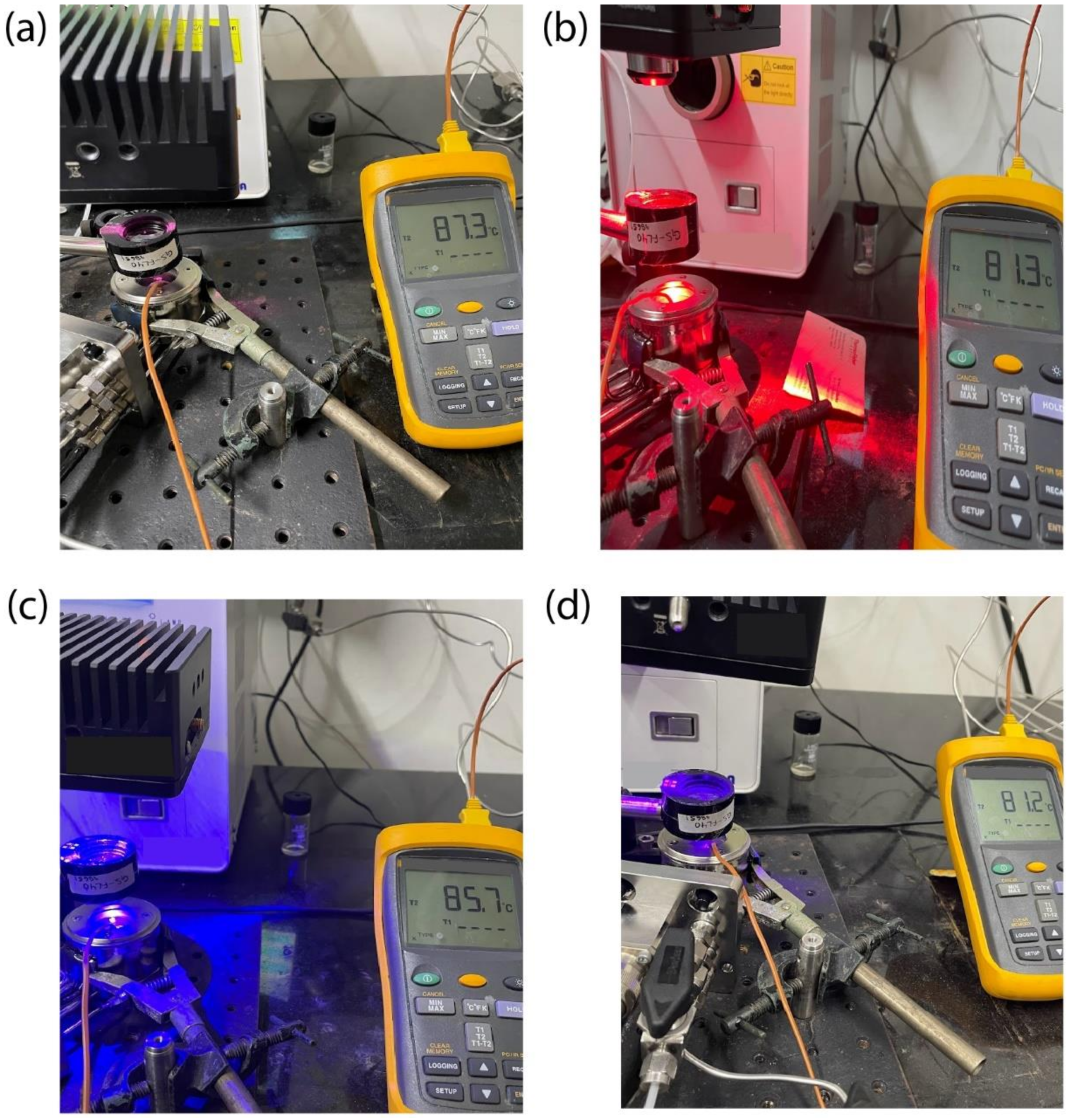

Figure S22. Measurement of Ts using external thermocouple at different wavelengths of light (a) 808 nm; (b) 637 nm; (c) 447 nm; (d) 405 nm with the optimized reactant gas flow- 30 mL min^{-1} C_2H_2 (10 % in Ar), 60 mL min^{-1} C_2H_4, 15 mL min^{-1} H_2, 5 mL min^{-1} air at 1 bar pressure and light intensity of 2.7 W cm^{-2}.

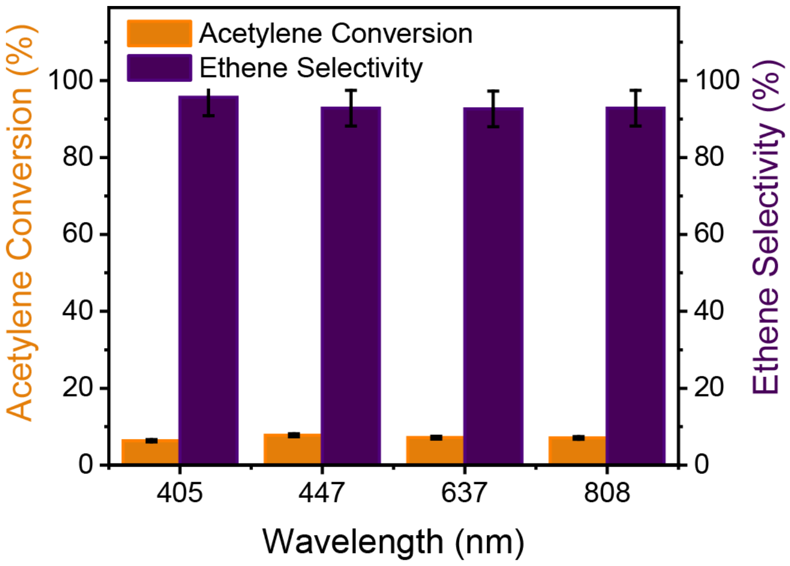

Figure S23. Acetylene conversion and ethene selectivity for acetylene semi-hydrogenation in excess ethene over DPC/RuPt-10-Calc at different wavelengths (at 2.7 W cm^{-2}) (Flow: 3 % acetylene in feed, H_2:C_2H_2=5:1, C_2H_4:C_2H_2=20:1 and 1320000 mL g^{-1} h^{-1} GHSV).

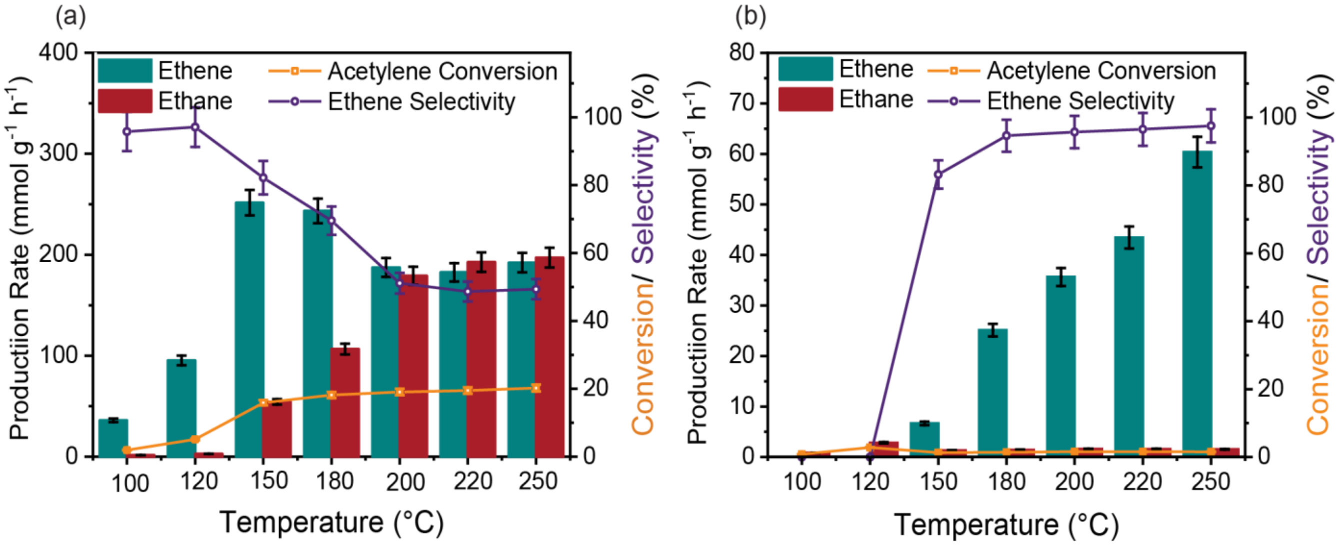

Figure S24. Acetylene semi-hydrogenation in excess ethene over (a) DPC/Pt -1-Calc; (b) DPC/Ru-9-Calc at different temperatures in the dark (Flow: 3% acetylene in feed, H_2:C_2H_2=5:1, C_2H_4:C_2H_2=20:1 and 1320000 mL g^{-1} h^{-1} GHSV).

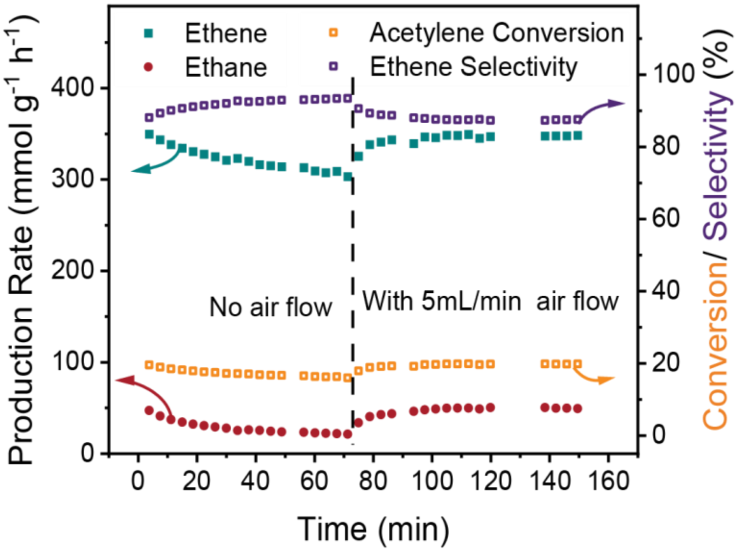

Figure S25. Acetylene semi-hydrogenation in excess ethene over DPC/RuPt-10-Calc with and without air (3% acetylene in feed, 5:1 H_2: C_2H_2 ratio, C_2H_4:C_2H_2=20:1 and 1320000 mL g^{-1} h^{-1} GHSV, visible light illumination (400–1100 nm) with light intensity of 2.7 W cm^{-2}).

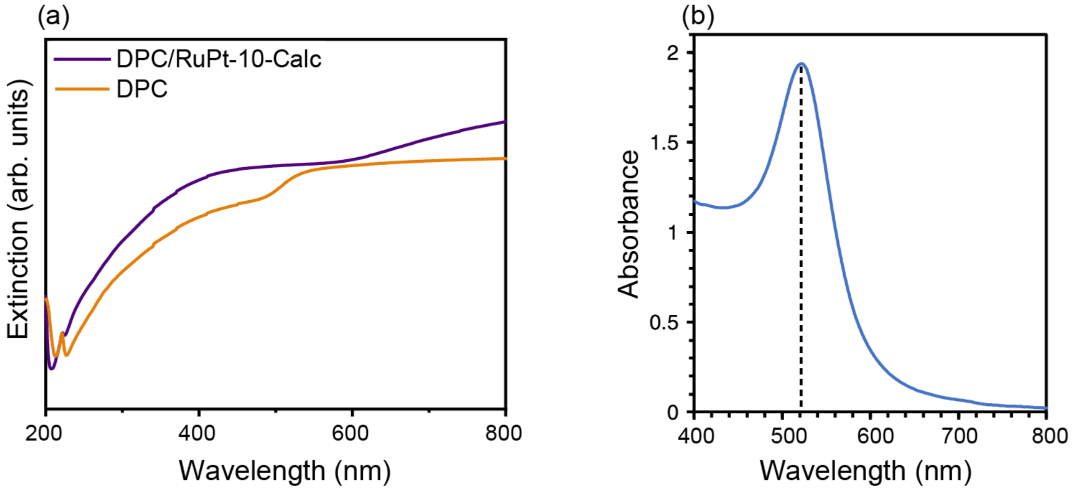

Figure S26. (a) UV-Vis extinction spectra of DPC and DPC/RuPt as a dispersion form in ethanol showing broadband absorption in the visible range; (b) UV−vis extinction spectrum of a colloid of the Au NPs used for the preparation of the photocatalyst film. The spectrum exhibits a localized surface plasmon resonance (LSPR) band centered around 520 nm, as indicated by the dotted line (reproduced from reference-2, Copyright 2019 Springer Nature).

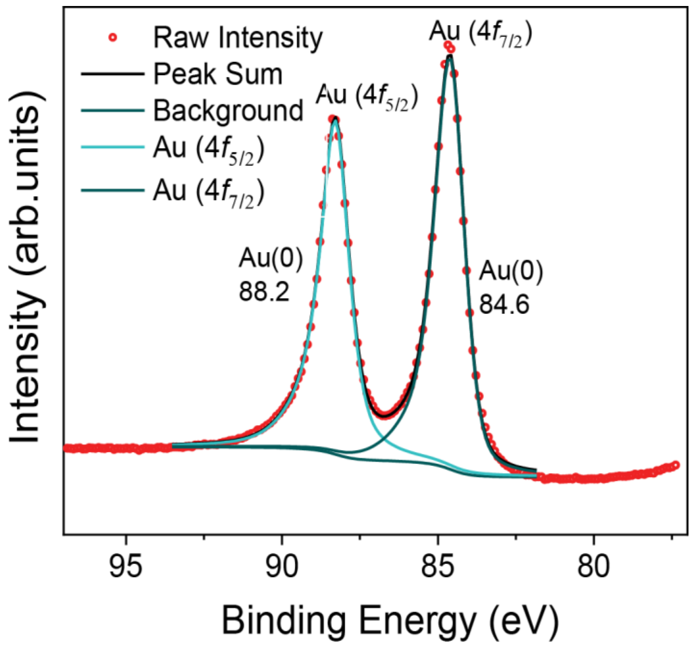

Figure S27. XPS analysis of DPC/RuPt-10-Calc-Au (4*f*) showing Au in the elemental state.

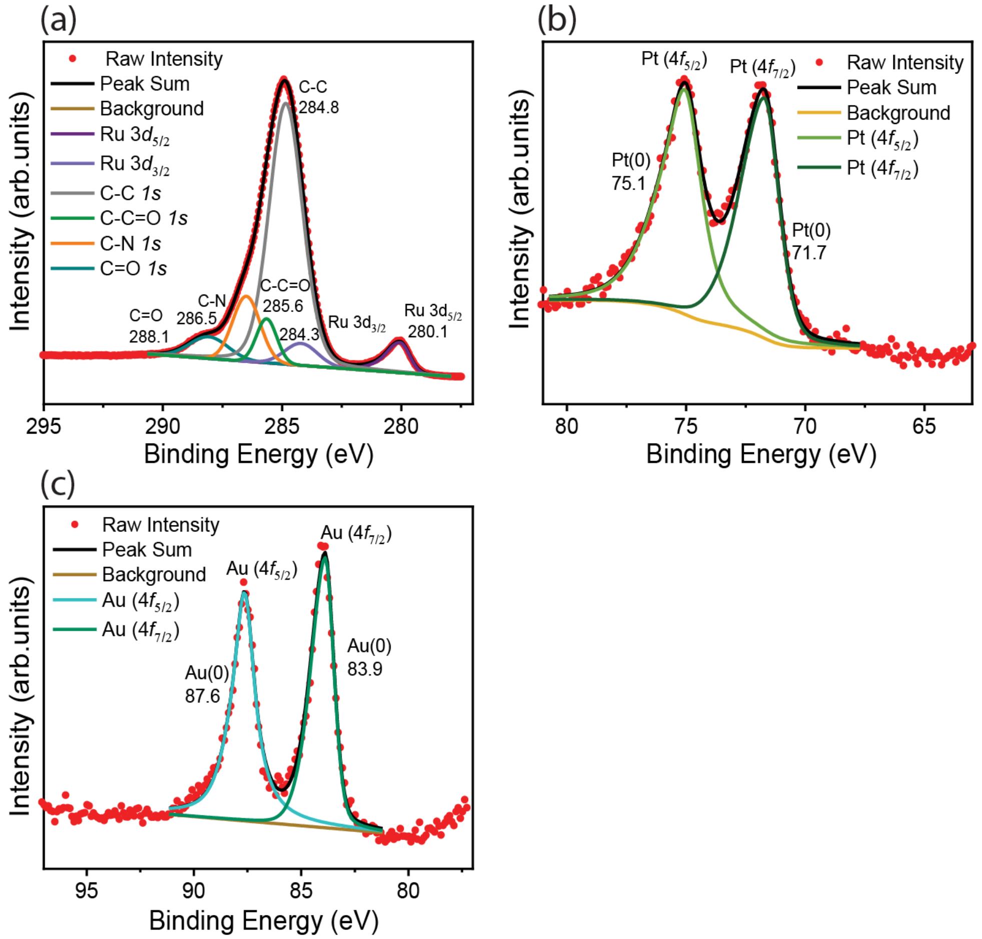

Figure S28. XPS analysis of DPC/RuPt-10-ASP (a) Ru (3*d*); (b) Pt (4*f*); (c) Au (4*f*).

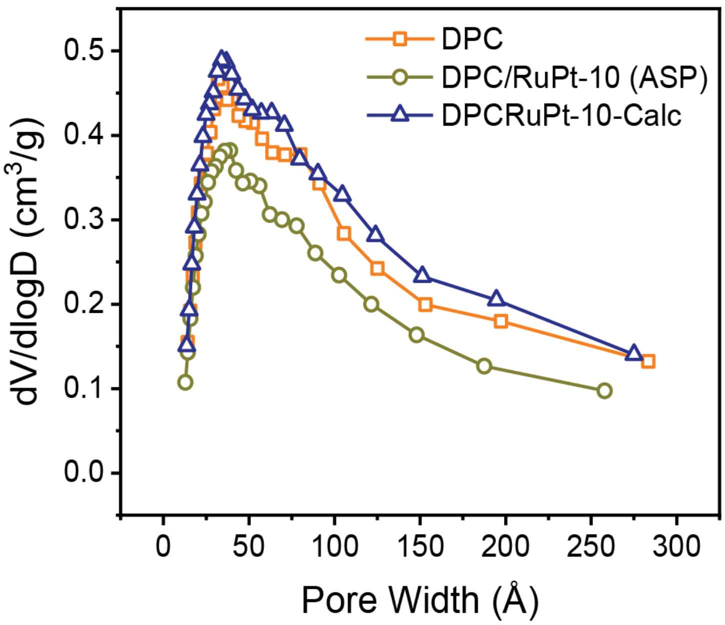

Figure S29. Pore size distribution of different catalysts using nitrogen sorption isotherms.

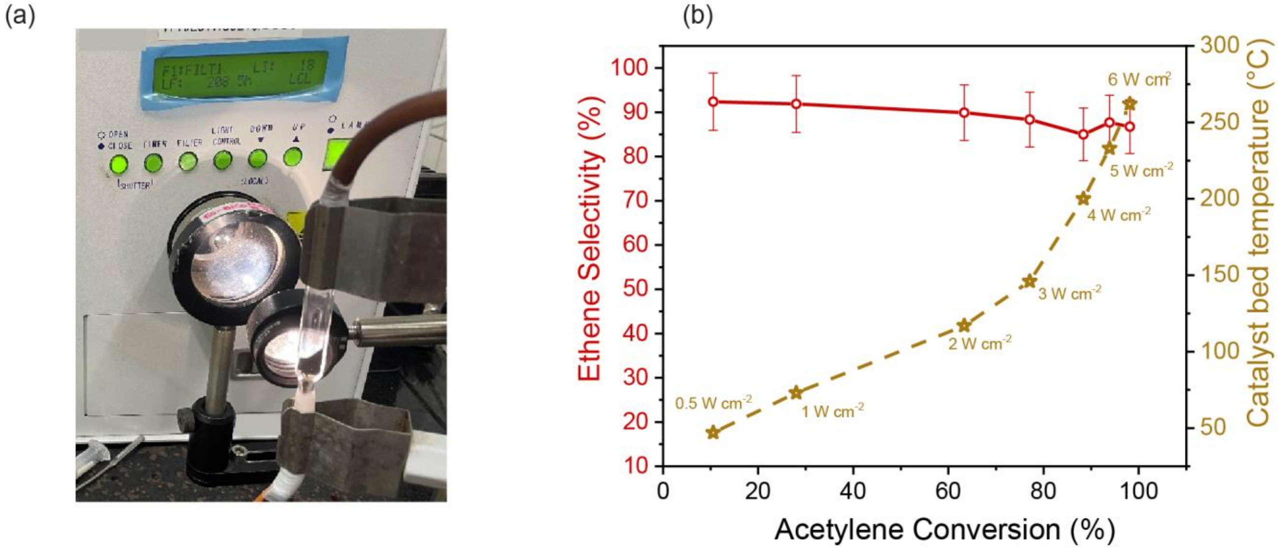

Figure S30. (a) Quartz flat cell flow reactor setup employed to increase the illuminated catalyst area; (b) acetylene conversion and ethene selectivity trend showing high selectivity being maintained at high conversion. The highest conversion was achieved with a total flow of 9 mL min^{-1}, $C_2H_2/C_2H_4/H_2$/Ar/Air=0.2/4/2/1.8/1 ml min^{-1} and AM1.5 illumination at 1 bar pressure. Quartz flat cell flow reactor dimensions: the internal gap is 0.5 mm in the flat section, length- 50 mm, width- 8 mm.

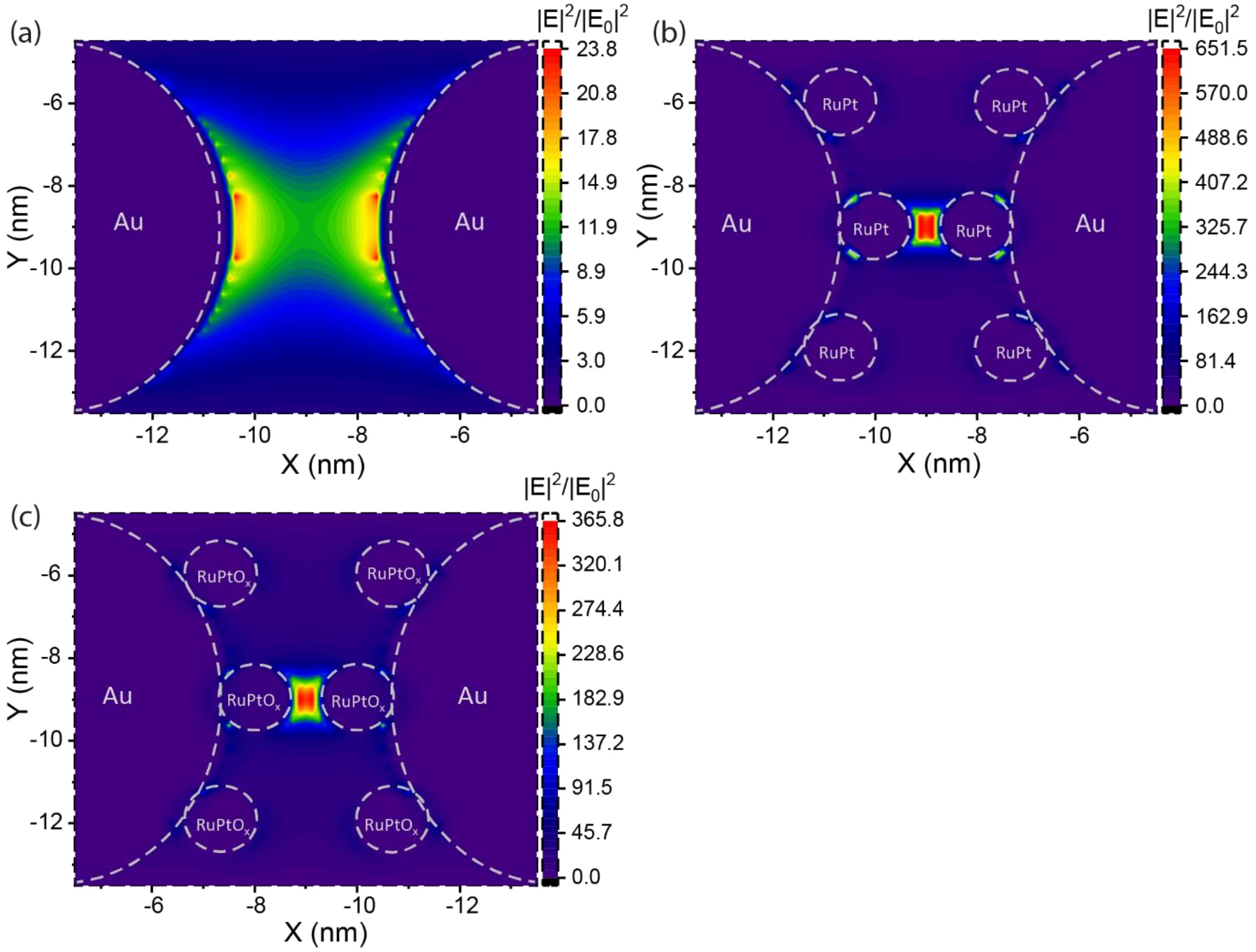

Figure S31. Electric field enhancement in (a) DPC; (b) DPC/RuPt; (c) DPC/$RuPtO_x$ using FDTD simulations.

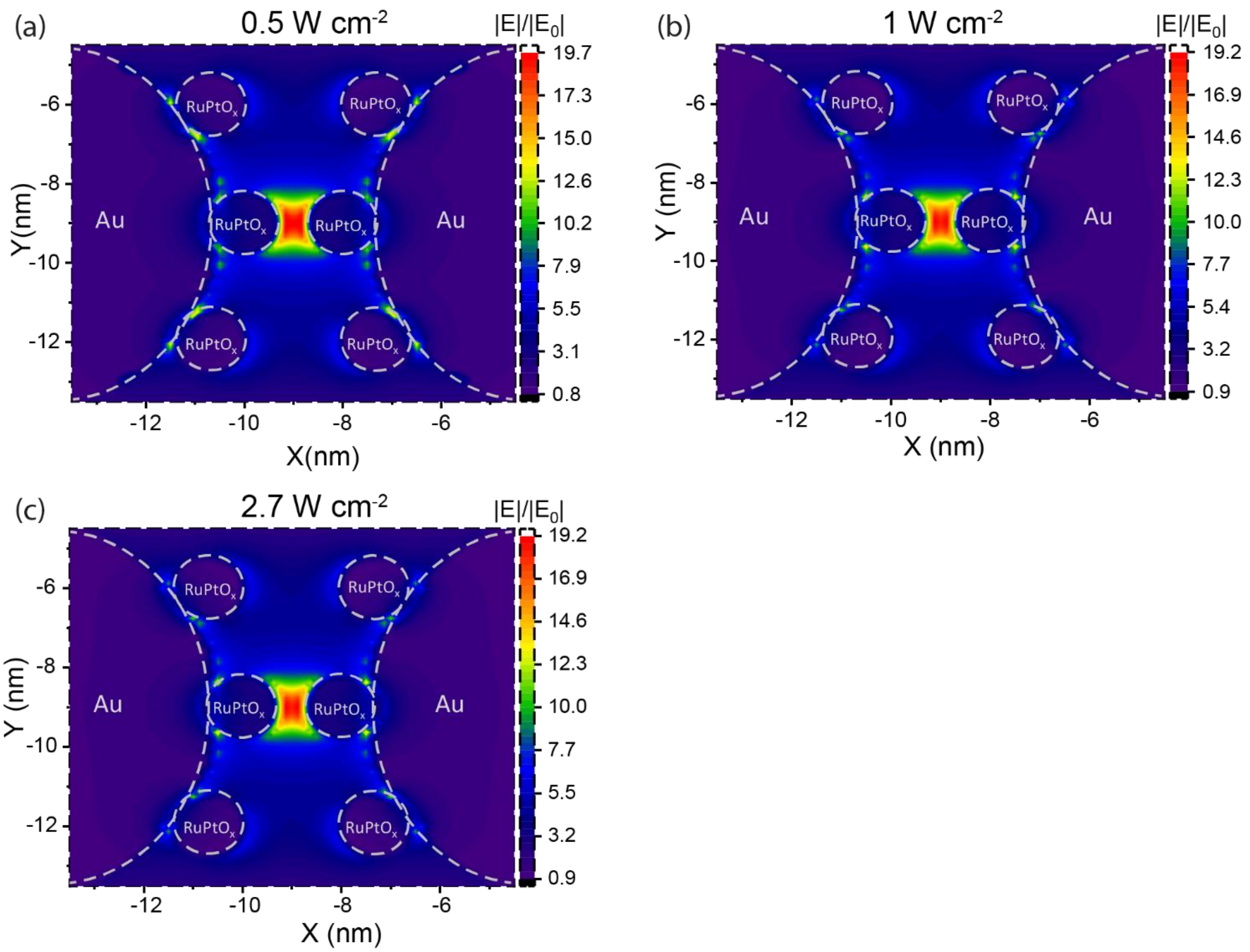

Figure S32. Electric field enhancement in DPC/$RuPtO_x$ at different light intensities, (a) 0.5 W cm^{-2}; (b) 1 W cm^{-2}; (c) 2.7 W cm^{-2} using FDTD simulations.

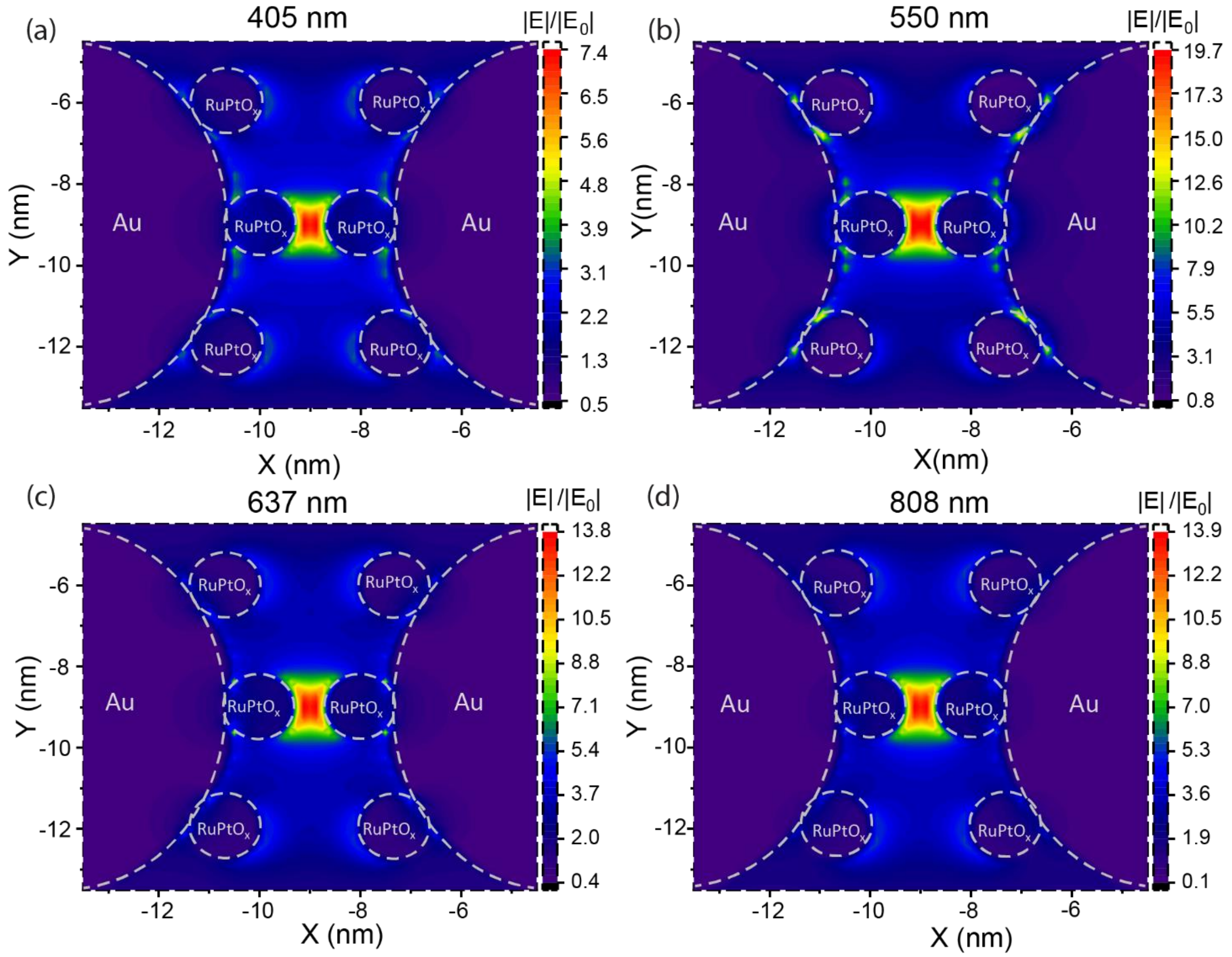

Figure S33. Electric field enhancement in DPC/$RuPtO_x$ at different wavelengths, (a) 405 nm; (b) 550 nm; (c) 637 nm; (d) 808 nm using FDTD simulations.

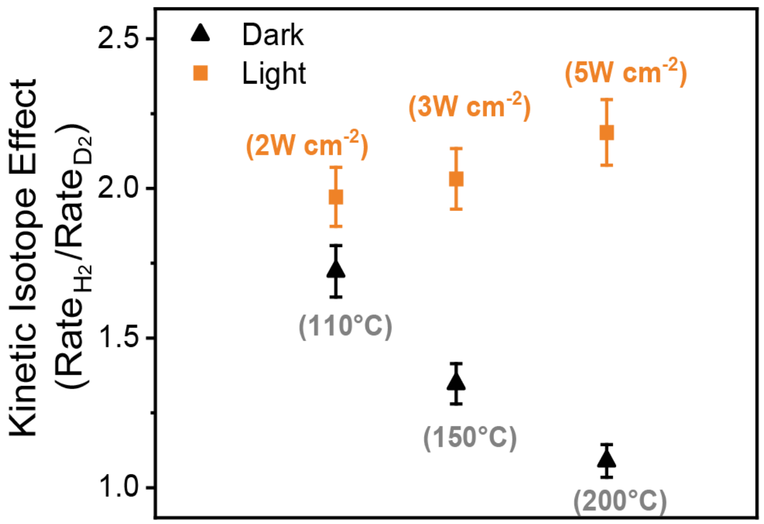

Figure S34. KIE dependence on light intensity and temperature (in the dark).

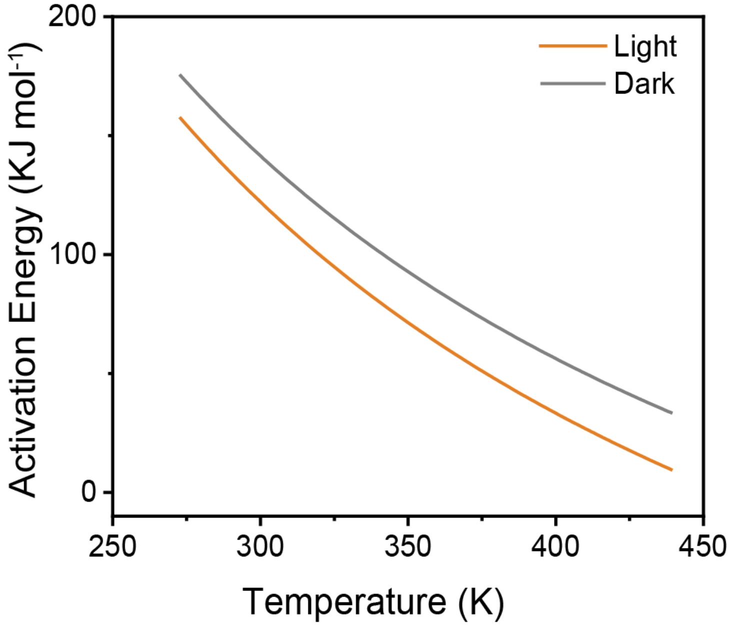

Figure S35. Activation energy vs. temperature plot for acetylene semi-hydrogenation over DPC/RuPt-10-Calc calculated using the Arrhenius equation.

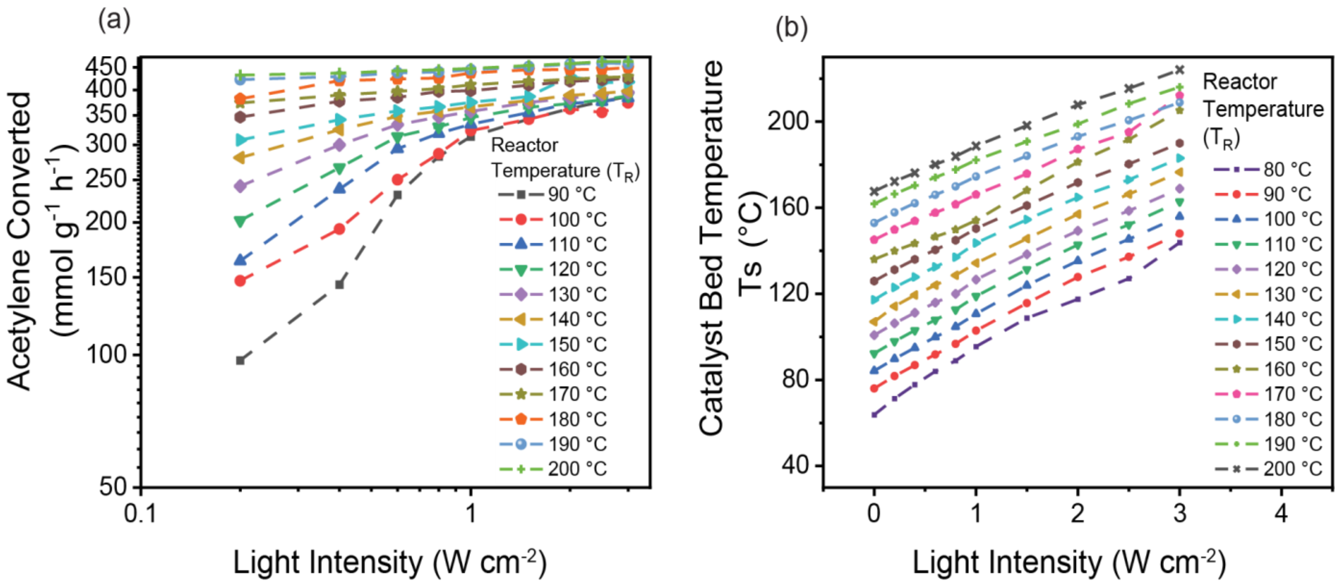

Figure S36. Intensity-temperature study for acetylene semi-hydrogenation in excess ethene over DPC/RuPt -10-Calc; (a) moles of acetylene converted (log scale) v/s light intensity (log scale) used at different reactor temperatures (T_R); (b) Variation of catalyst bed temperature (Ts) with different light intensities at different reactor temperatures with 3 % acetylene in feed, 5:1 H_2:C_2H_2 ratio, C_2H_4:C_2H_2=20:1 and 1320000 mL g^{-1} h^{-1} GHSV, visible light illumination (400–1100 nm).

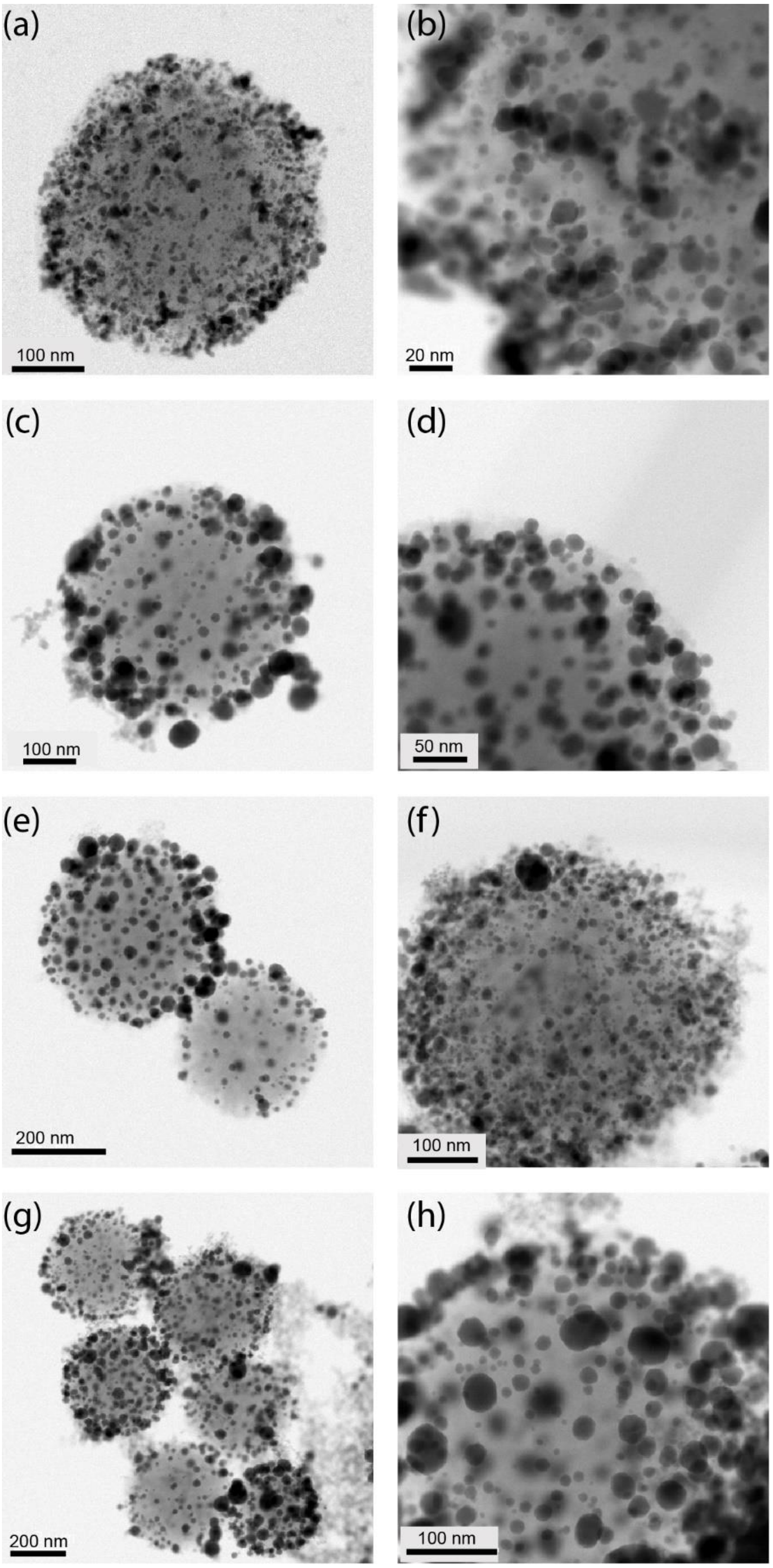

Figure S37. BF-STEM images of (a-b) DPC/RuPt-10-ASP; (c-d) DPC/RuPt-10-Calc; (e-f) DPC/RuPt-10-spent (with air); (g-h) DPC/RuPt-10-spent (without air).

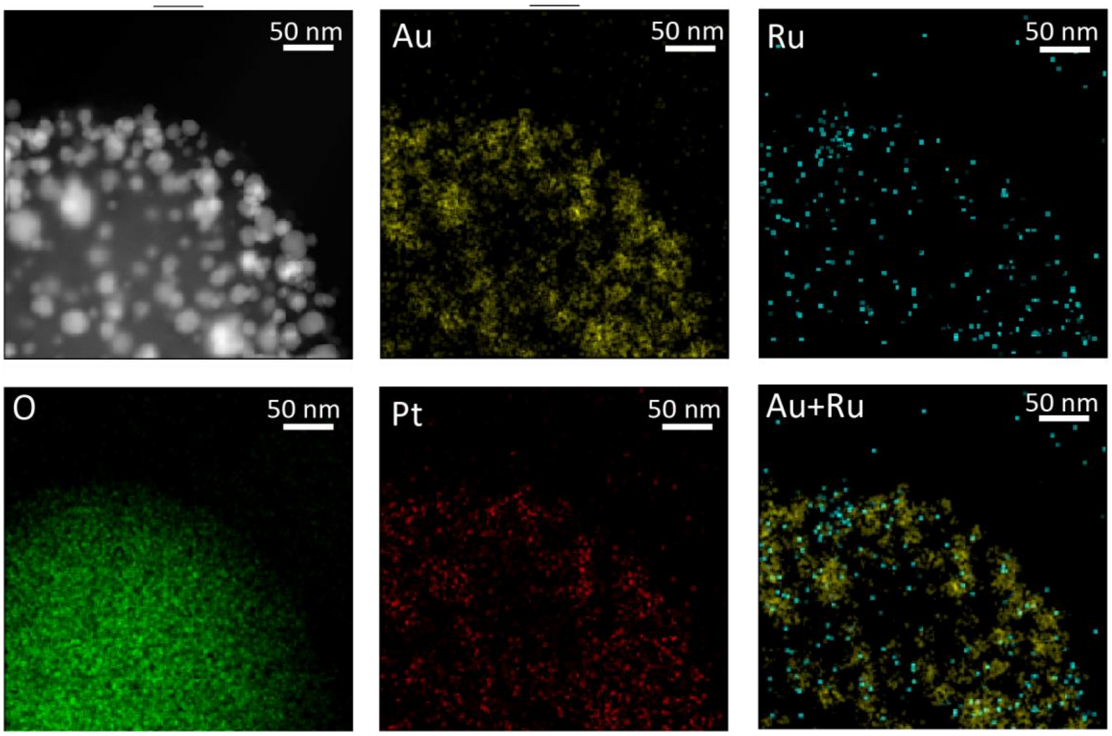

Figure S38. HAADF-STEM image and EDS elemental maps for DPC/RuPt-10-Calc.

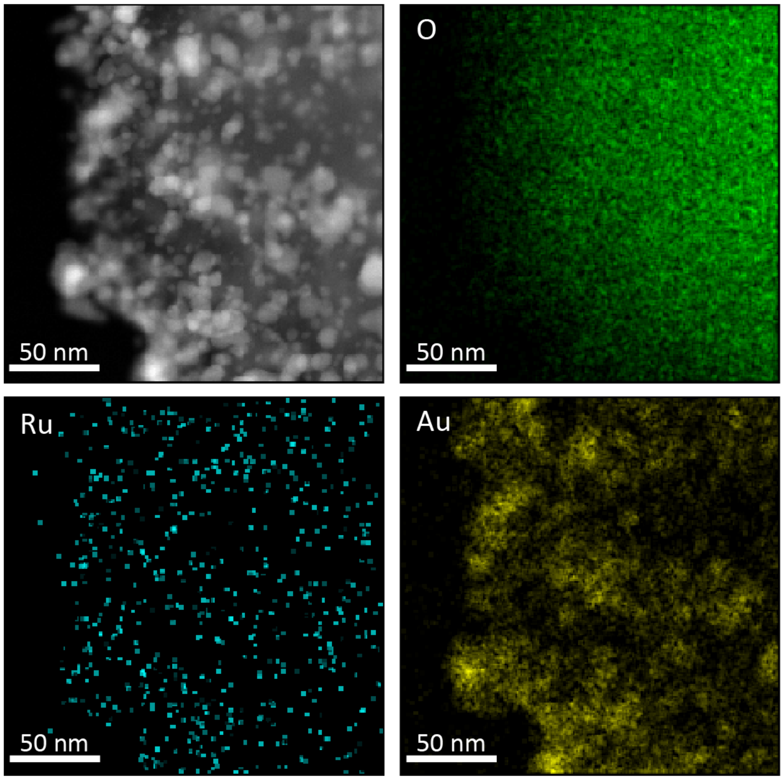

Figure S39. HAADF-STEM image and EDS elemental maps for DPC/RuPt-10-ASP.

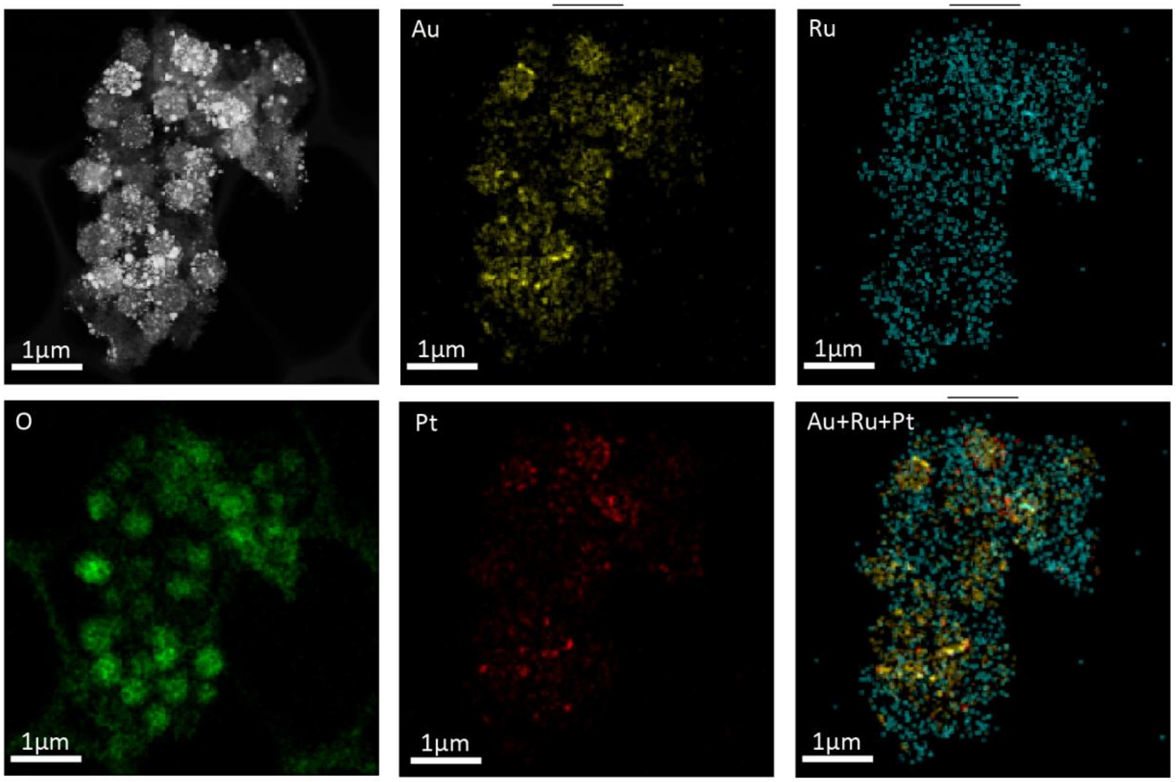

Figure S40. HAADF-STEM image and EDS elemental maps for DPC/RuPt-10-spent (with air).

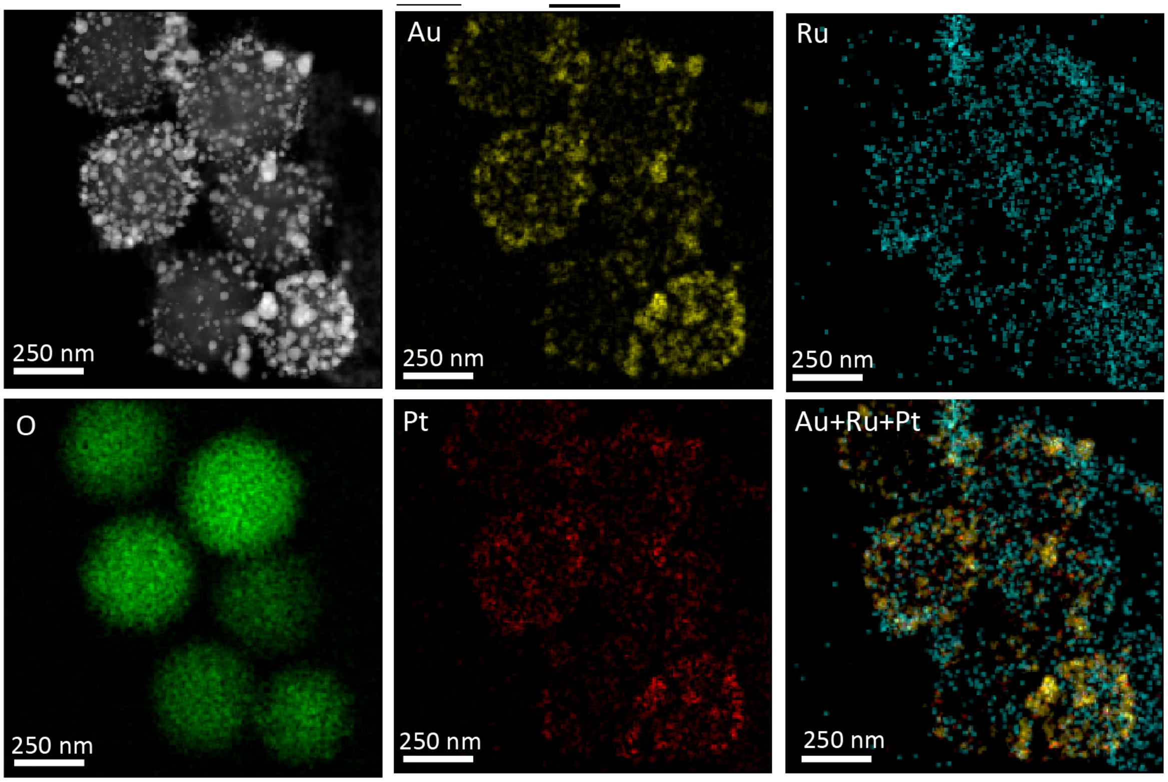

Figure S41. HAADF-STEM image and EDS elemental maps for DPC/RuPt-10-spent (without air).

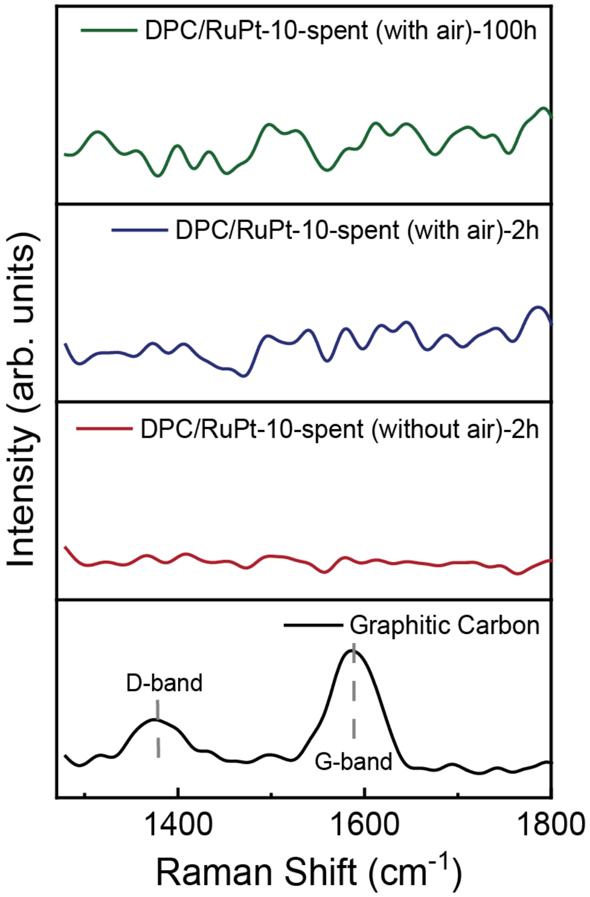

Figure S42. Raman spectroscopic analysis of different spent catalysts to determine graphitic carbon formation.

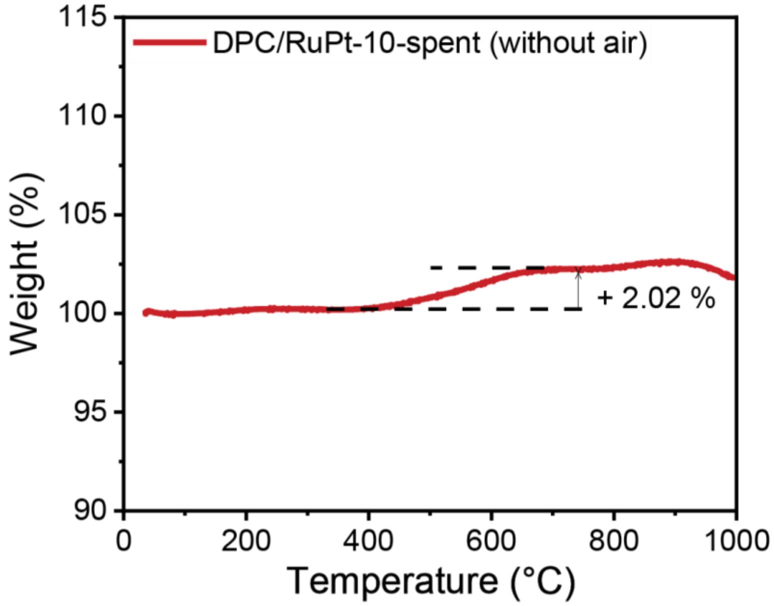

Figure S43. Thermogravimetric (TGA) analysis of DPC/RuPt-10-spent (without air) for 3 h in airflow (40 mL min^{-1}) from 30 °C to 1000 °C showing no coke formation and Ru oxidation.

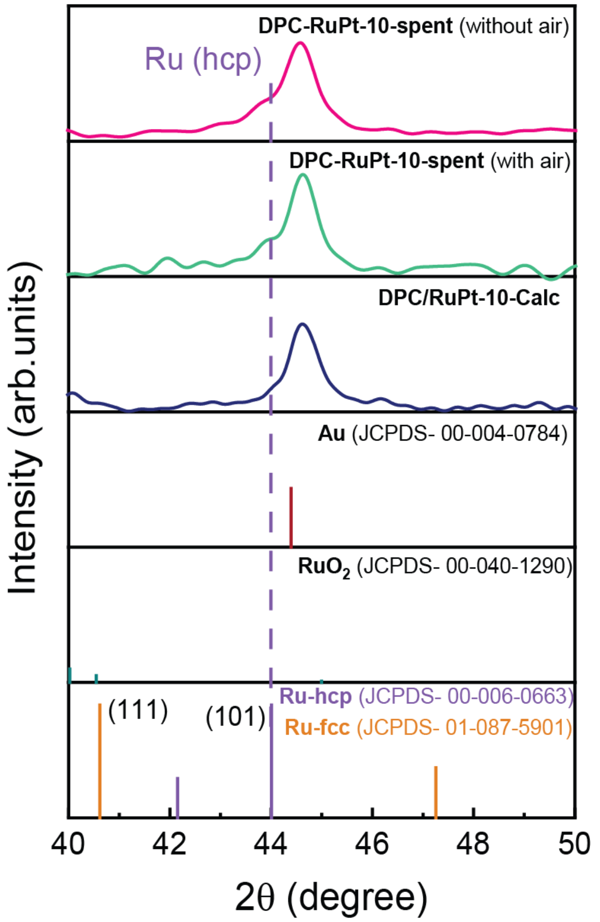

Figure S44. PXRD analysis of spent and calcined samples showing the formation of Ru-hcp during the reaction.

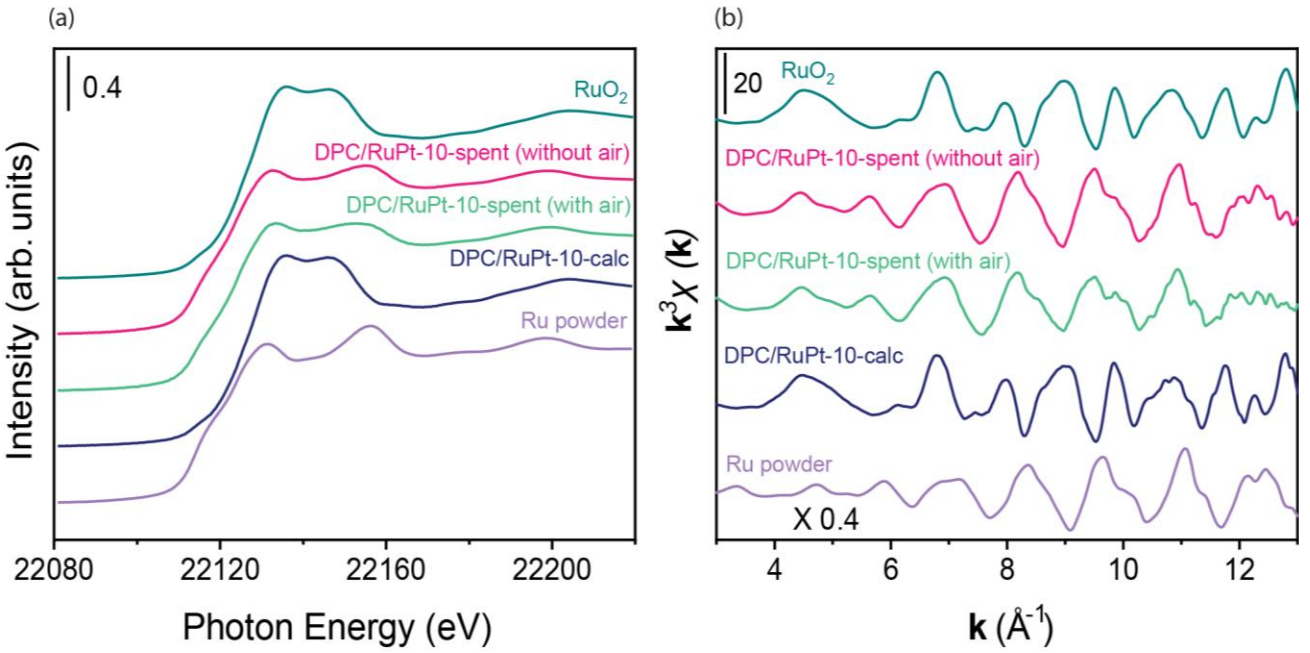

Figure S45. (a) Ru K-edge XANES spectra; (b) Ru K-edge EXAFS oscillations of each sample.

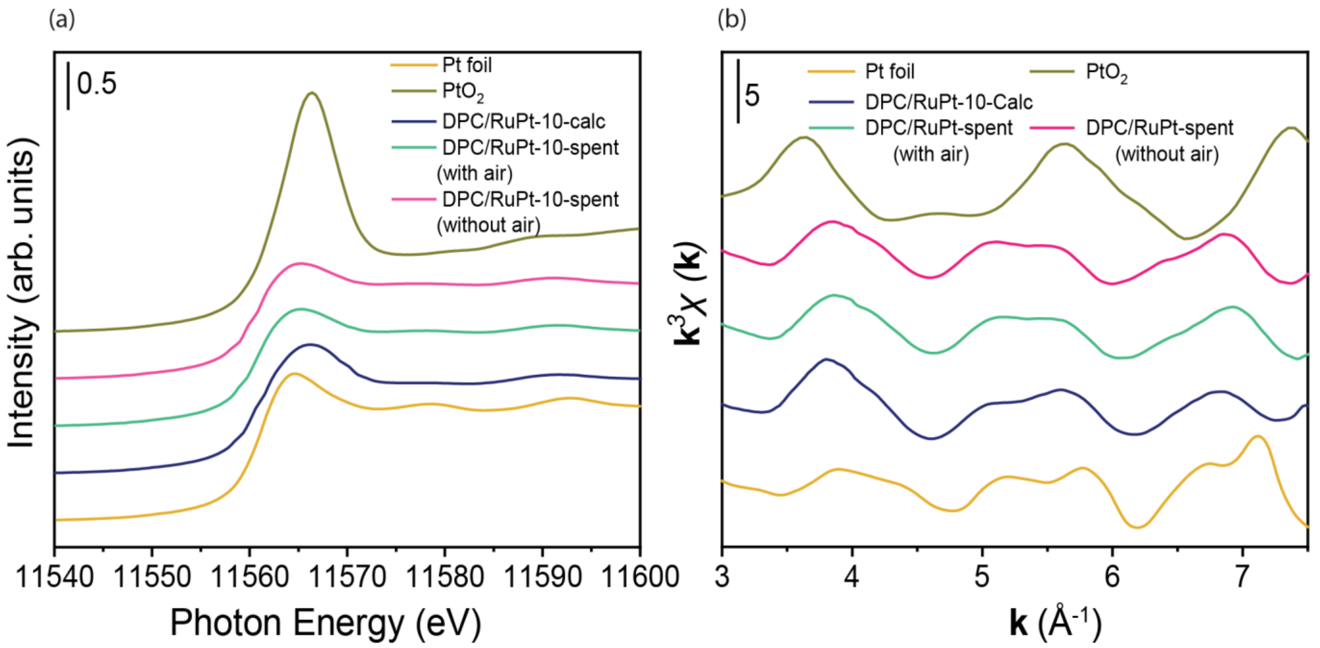

Figure S46. (a) Pt L_3-edge XANES spectra; (b) Pt L_3-edge EXAFS oscillations of each sample. Fourier transform (FT) of EXAFS oscillations of Pt-L_3 edge could not be analyzed due to strong overlap with Au-L_3 edge.

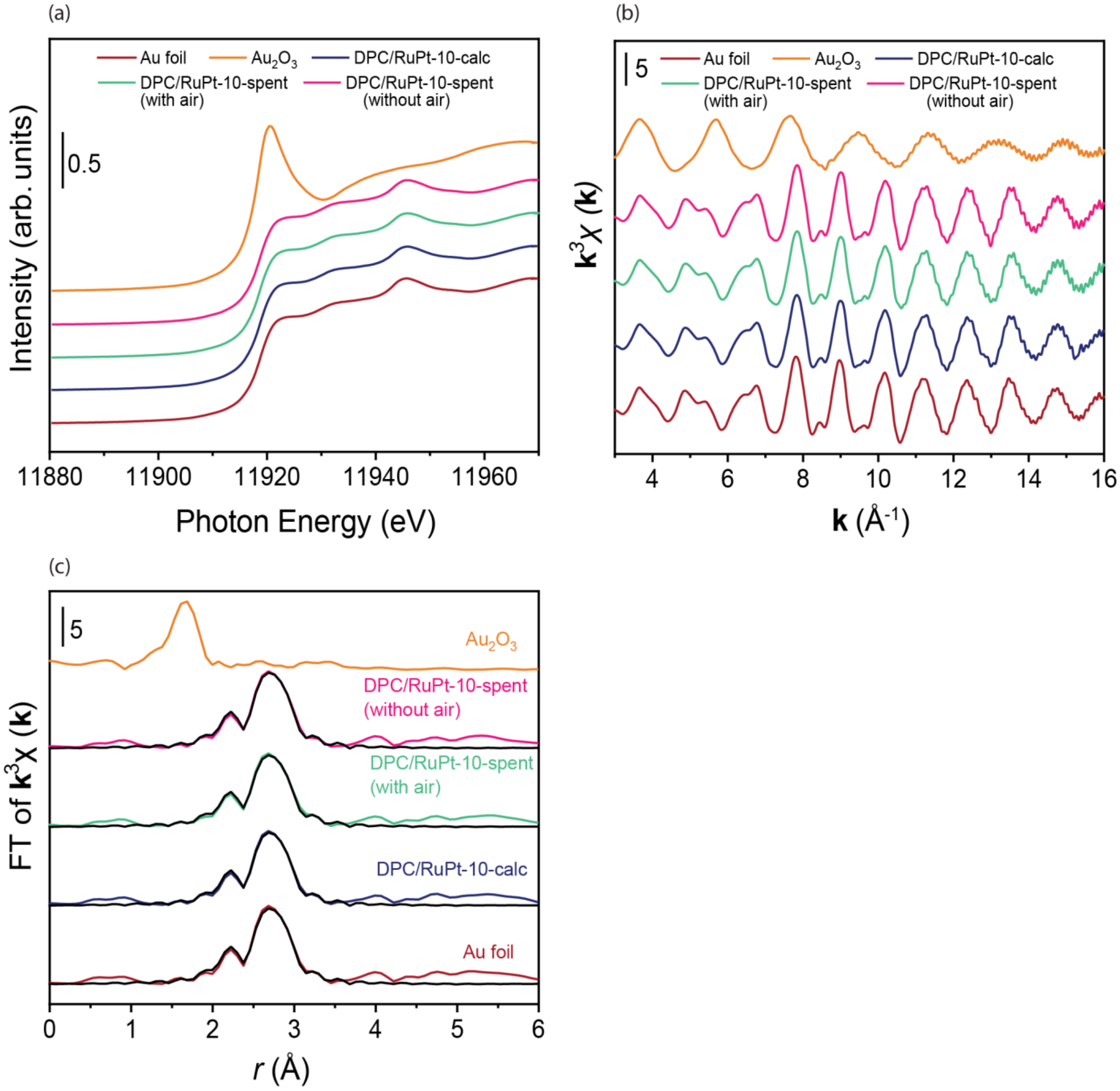

Figure S47. (a) Au L_3-edge XANES spectra; (b) Au L_3-edge EXAFS oscillations; (c) Fourier transform (FT) of Au L_3-edge EXAFS of each sample.

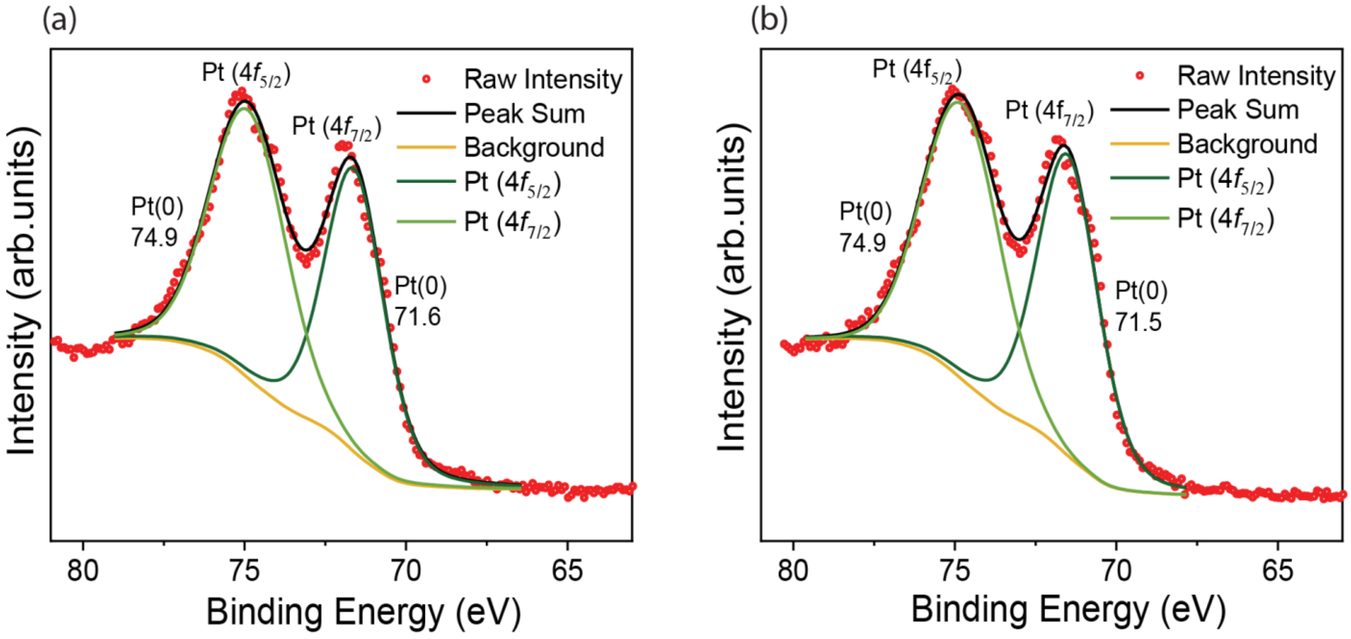

Figure S48. XPS analysis (Pt 4*f*), (a) DPC/RuPt-10-spent (with air); (b) DPC/RuPt-10-spent (without air).

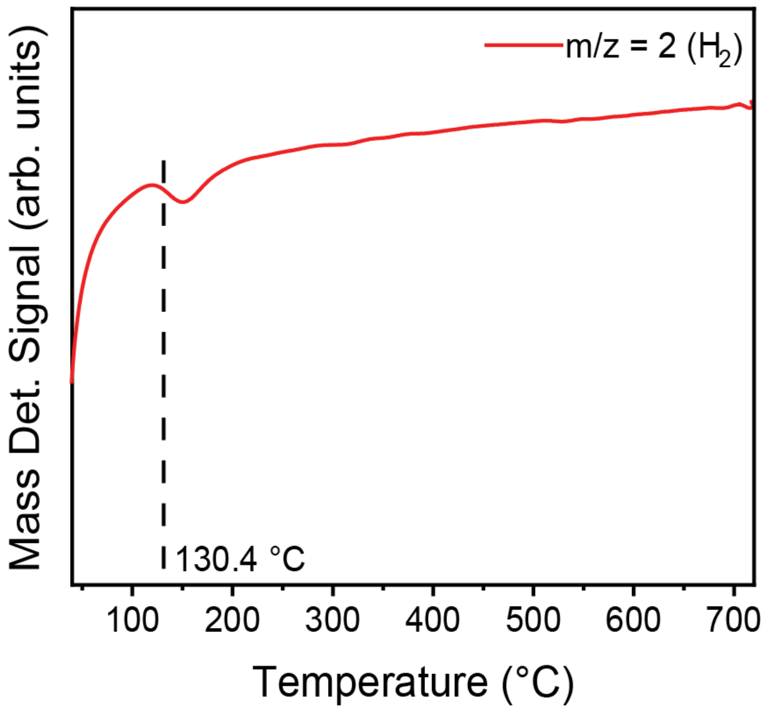

Figure S49. H_2-TPR analysis of DPC/RuPt-10-Calc with 20 % H_2 flow showing reduction at ~130 °C.

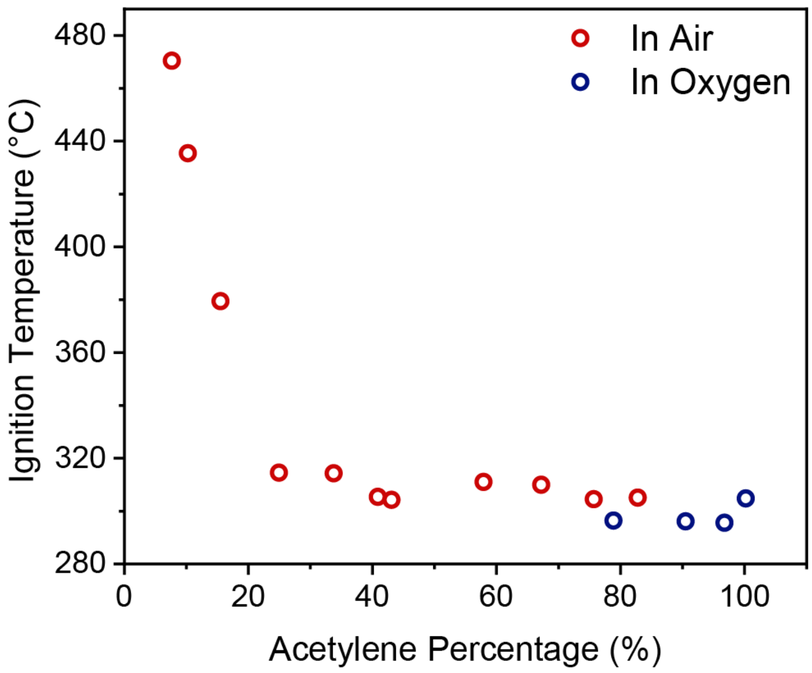

Figure S50. Dependence of ignition temperature of acetylene on its concentration in the atmosphere (plotted as per data in reference-3)

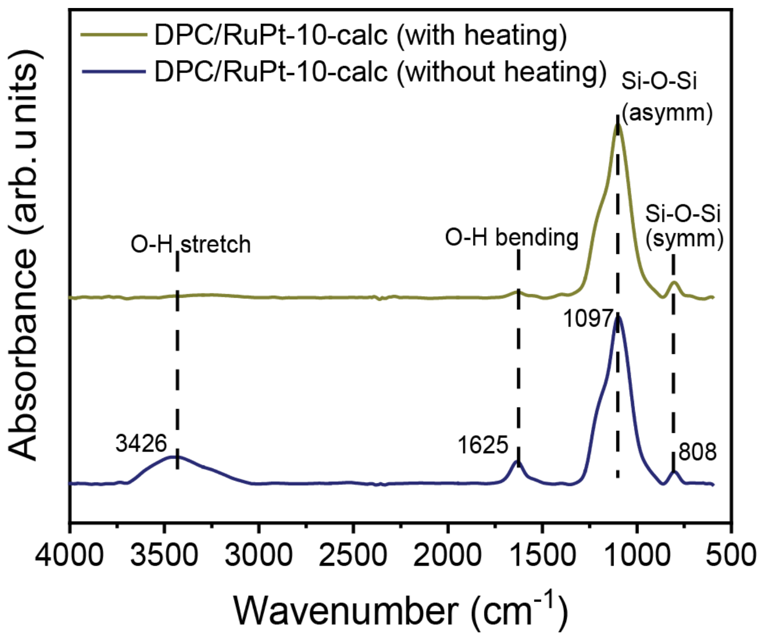

Figure S51. FTIR spectra of DPC/RuPt-10-Calc with and without heating at 100 °C in Ar for 60 min to remove adsorbed moisture.

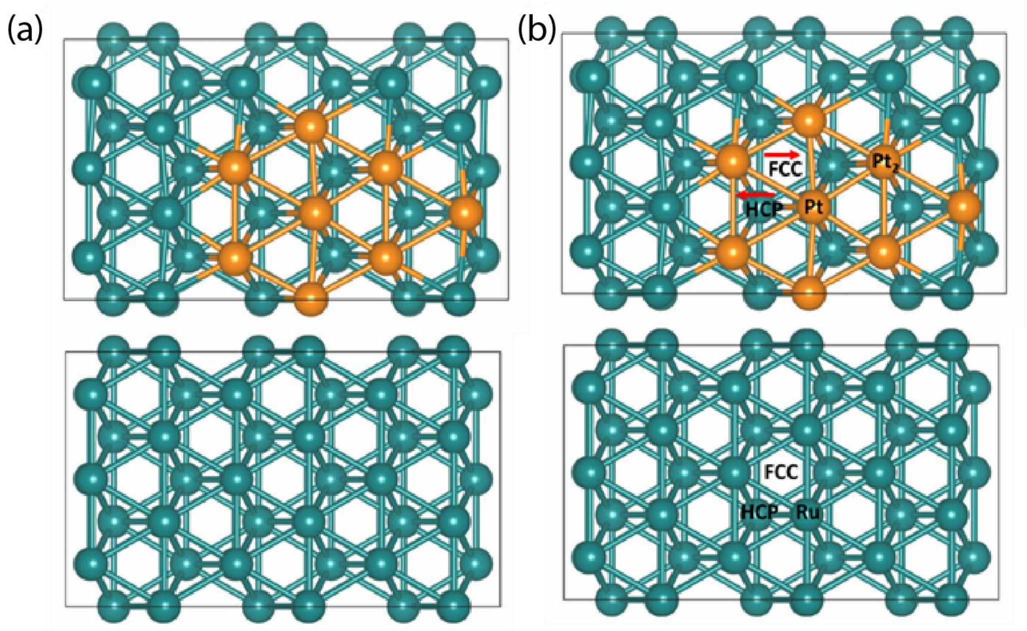

Figure S52. (a) Schematic structure of (110) surface of RuPt (top) and Ru (bottom). Dark cyan and orange colours represent Ru and Pt atoms, respectively; (b) Possible adsorption sites of H atom on RuPt (top) and Ru (bottom) 110 surface. The red arrows indicate a shift in adsorption sites after relaxation.

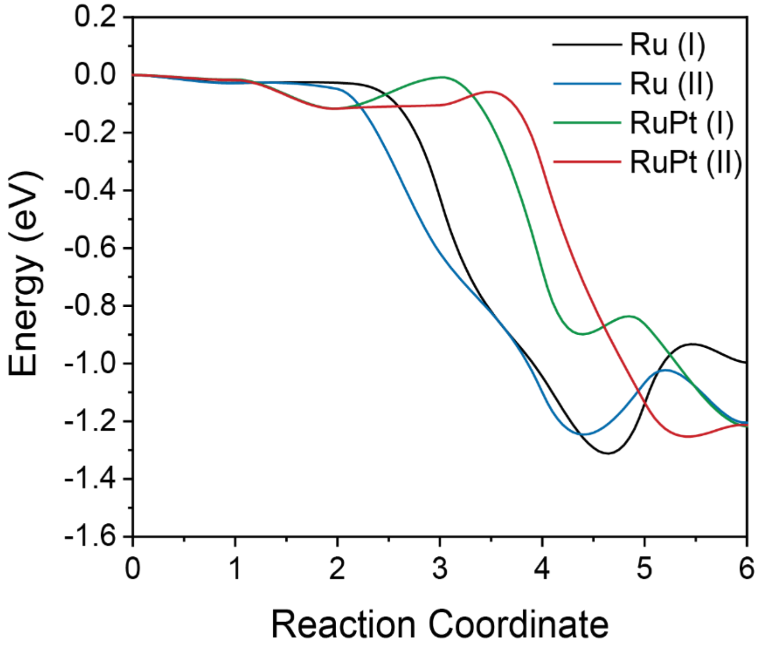

Figure S53. Calculated reaction energies of different dissociation paths of H_2 molecule for Ru surface: Path I: H_2 → $H_{(HCP)}$ + $H_{(FCC)}$, Path II: H_2 → $H_{(HCP)}$ + $H_{(HCP)}$ and for the RuPt surface: Path I: H_2 → $H_{(HCP\text{-}Bridge)}$ + $H_{(Pt)}$, Path II: H_2 → $H_{(HCP\text{-}Bridge)}$ + $H_{(FCC\text{-}Bridge)}$.

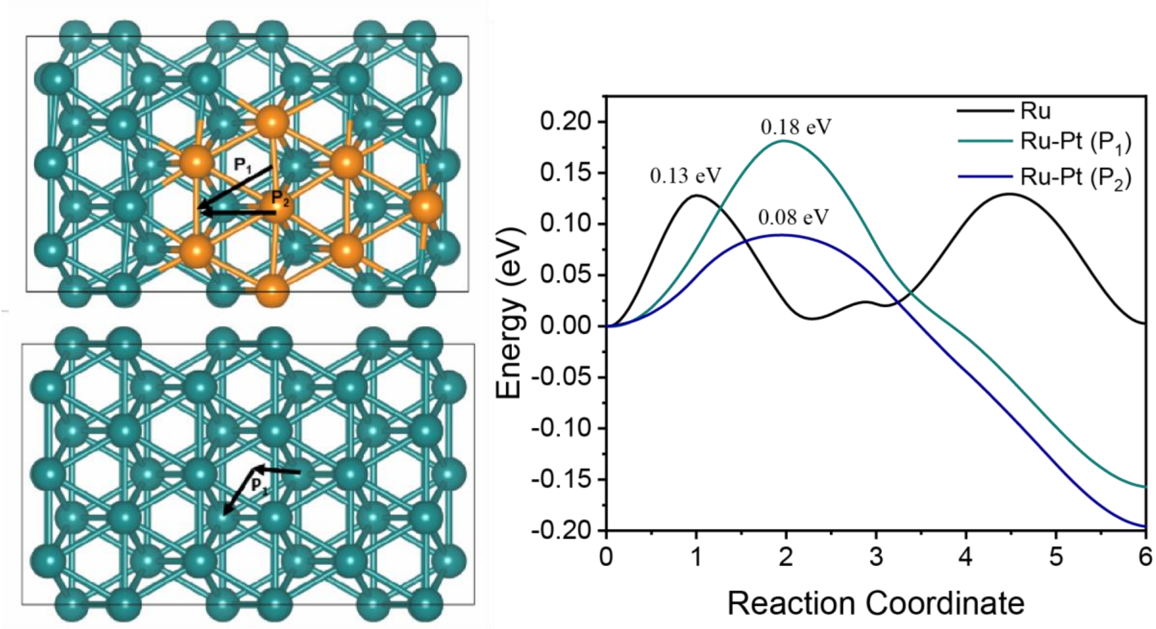

Figure S54. Migration paths of H atom on RuPt (top-left) and Ru (bottom-left). Calculated corresponding reaction energies are shown on the right.

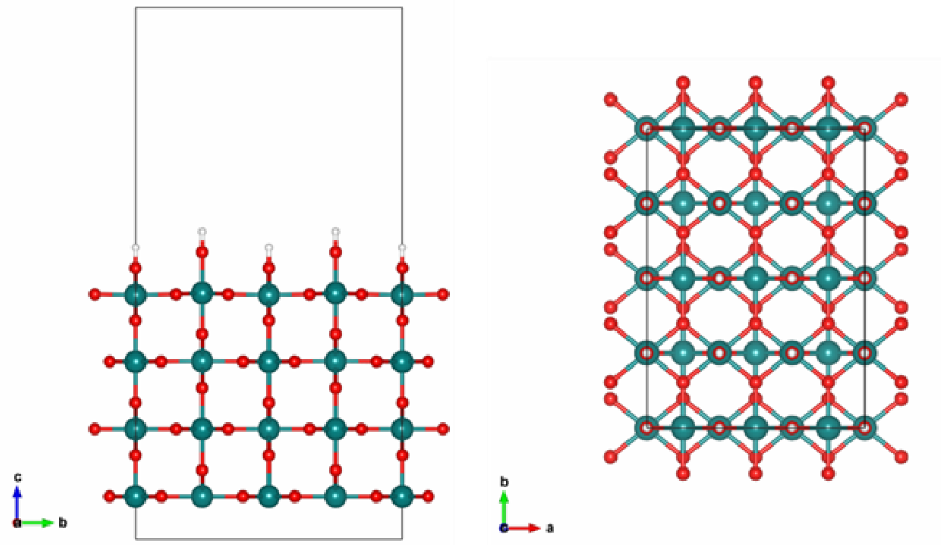

Figure S55. Side view (left) and top view (right) hydrogen terminated RuO_2 (110) surface. The white atom represents the H atom.

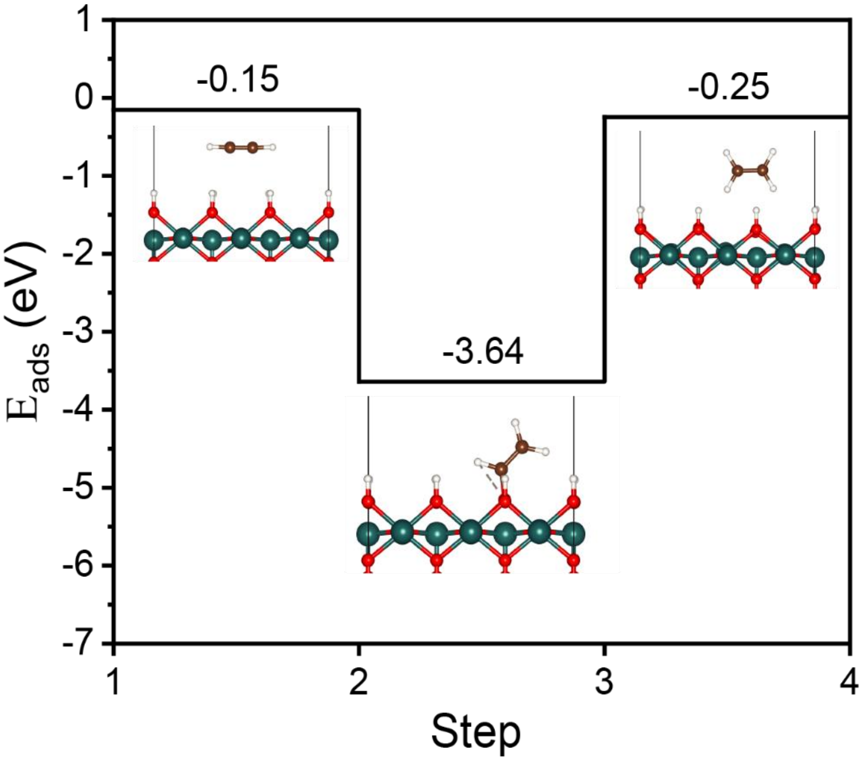

Figure S56. Calculated hydrogenation process of C_2H_2 to C_2H_4.

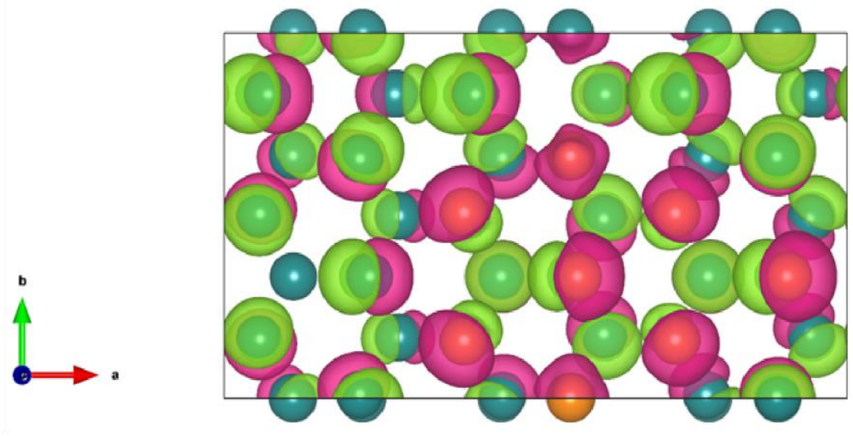

Figure S57. Calculated charge density difference between Ru and RuPt surface showing larger charge accumulation on Pt. Pink and Green colours indicate charge accumulation and depletion, respectively. Blue-Ru and Orange-Pt.

Table S1. Effective metal content in Ru and Pt doped Ru clusters determined by ICP-MS analysis.

Sample	Ru (wt%)	Pt (wt%)	Total metal content (wt%)
Ru	1.97	–	1.97
RuPt	1.78	0.29	2.07

Table S2. Elemental composition of different catalysts by SEM-EDS.

Entry	Sample	Si (wt%)	O (wt%)	Au (wt%)	Ru (wt%)	Pt (wt%)
1	DPC/RuPt-3-Calc	41.5 ± 7.0	16.9 ± 3.0	36.8 ± 10.1	4.7 ± 1.4	–
2	DPC/RuPt-5-Calc	49.1 ± 11.6	15.6 ± 3.2	29.1 ± 11.9	6.0 ± 2.8	–
3	DPC/RuPt-10-Calc	59.9 ± 10.6	12.5 ± 3.3	16.9 ± 7.6	10.5 ± 4.1	0.2 ± 0.4
4	DPC/RuPt-20-Calc	50.1 ± 9.3	11.0 ± 2.1	19.4 ± 6.6	18.3 ± 3.3	1.07 ± 0.8

Table S3. BET surface areas and BJH pore volumes of different catalysts.

Sample	BET Surface Area (m^2/g)	BJH Pore Volume (cm^3/g)
DPC	356	0.41
DPC-RuPt-ASP	355	0.34
DPC-RuPt-Calc	415	0.45

Standard error in measurement : ±10% in BET Surface area, ± 0.02 in Pore Volume

Table S4. Comparison of the best reported non-plasmonic catalytic systems employed for semi-hydrogenation of acetylene.

Entry	Catalyst (loading, weight)	Temperature (°C)	Feed Composition in vol. % (total flow)	Reactor Type	Ethene Production Rate (mmol g^{-1} h^{-1})	Acetylene Conversion (%)	Ethene Selectivity (%)	Ref.
1	DPC/RuPt-10-Calc (10 wt%, 5 mg)	T_R-75<br>Ts-132 with light 2.7 W cm^{-2} 400-1100 nm	$C_2H_2/C_2H_4/H_2$/Ar/Air = 2.72/54.5/13.6/24.5/4.5 (110 mL min^{-1})	Fixed bed flow reactor with quartz window (crucible i.d- 6mm)	320	18	88	*This Work*
2	DPC/RuPt-10-Calc (10 wt%, 20 mg)	Ts- 262<br>light 6 W cm^{-2}<br>AM 1.5	$C_2H_2/C_2H_4/H_2$/Ar/Air = 2.2/44.4/22.2/20/11.1 (9 mL min^{-1})	Quartz flat cell flow reactor (internal gap is 0.5 mm in the flat section, length-50 mm, width-8 mm)	31	97	87	*This Work*
3	Pd/ZnO (1 wt%, 10 mg)	80	$C_2H_2/C_2H_4/H_2$/He = 2/40/20/38 (30 mL min^{-1})	Fixed bed quartz microreactor (i.d. 4 mm)	131.5	92	89	4
4	PdIn/$MgAl_2O_4$ (2 wt%, 25 mg)	90	$C_2H_2/C_2H_4/H_2$/He = 0.5/50/5/44.5 (120 mL min^{-1})	Quartz bed flow reactor	54.9	95	90	5
5	Pd-Pt/SiO_2 (1.5 wt%, 20 mg)	80	C_2H_2/H_2/He = 1.67/3.33/95 (60 mL min^{-1})	Fixed bed flow quartz reactor (1/4 inch)	52.0	97	40	6
6	Pd1/TiO_2 (SAC) (0.15 wt%, 15 mg)	120 (dark)<br>60 (light-167 mW cm^{-2}, UV-Vis)	$C_2H_2/C_2H_4/H_2$/He = 1/20/10/69 (45 mL min^{-1})	Quartz fixed-bed flow reaction chamber equipped with a quartz window (d = 35 mm)	48.2<br>12.0	100<br>25	65-50<br>65	7
7	$Pd_{1.0}/Bi_2O_3/TiO_2$ (Pd 2.3 wt%, Bi 4.9 wt%, 30mg)	44	$C_2H_2/C_2H_4/H_2$/He =1/20/20/59 (60 mL min^{-1})	Fixed bed vertical quartz reactor	43.8	91	90	8
8	Pd Single atom/N-graphene (1.04 wt%, 50 mg)	125 (Photothermal: 5.1 W cm^{-2} , UV-Vis)	$C_2H_2/C_2H_4/H_2$/Ar = 1/20/20/59 (60 mL min^{-1})	Flow reactor with a quartz window at the top for light irradiation. (Volume- 50 cc)	29.7	99	93	9
9	Pd/ND@G (0.11 wt%, 30 mg)	180	$C_2H_2/C_2H_4/H_2$/He =1/20/10/69 (30 mL min^{-1})	Quartz bed flow reactor	24.1	100	90	10
10	$Pd_{0.006}Cu/SiO_2$	160	$C_2H_2/C_2H_4/H_2$/He = 1/20/20/59	Quartz reactor	22.7	100	85	11

	(Pd 0.05wt%, Cu 4.96wt%, 30 mg)		(30 mL min^{-1})					
11	$Al_{13}Fe_4$ (20 mg)	200	$C_2H_2/C_2H_4/H_2/He$ = 0.5/50/5/44.5 (30 mL min^{-1})	Quartz glass plug-flow reactor (i.d.- 7 mm) catalyst bed supported by a quartz glass frit.	13.6	80	85	12
12	PdZn-1.2@ZIF-8C (0.7 wt%, 50 mg)	120	$C_2H_2/C_2H_4/H_2/Ar$ = 0.65/50/5/45 (40 mL min^{-1})	Fixed-bed quartz tubular reactor	9.4	85	80	13
13	Na-Ni@CHA (Na 6.3 wt%, Ni 3.5 wt%, 200 mg)	180	$C_2H_2/H_2/He$ = 1/16/83 (50 mL min^{-1})	Quartz fixed bed flow microreactor	6.0	100	90	14
14	$GaPd/Al_2O_3$ (Pd 0.005 wt%, 75 mg)	200	$C_2H_2/C_2H_4/H_2/He$ = 0.5/50/5/44.5 (30 mL min^{-1})	Plug flow reactor consisting of a quartz glass tube with a length of 300mm, i.d.- 7mm and a sintered glass frit to support the catalyst.	3.9	87	85	15
15	Ga_2O_3-Pd/Al_2O_3 (0.23 wt%, 50 mg)	100	$C_2H_2/C_2H_4/H_2/N_2$ = 0.3/33.1/0.6/66 (50 mL min^{-1})	Fixed-bed flow quartz tube reactor	1.5	20	95	16
16	Ni MoS/Al_2O_3 (0.5 wt%, 0.5 g)	125	$C_2H_2/C_2H_4/H_2/He$ = 0.15/15/3/82 (165 mL min^{-1})	Fixed-bed microreactor	1.19	100	90	17
17	Single-atom Pd (0.16 wt%, 1 g)	120	$C_2H_2/C_2H_4/H_2/He$ = 0.5/50/5/44.5 (20 mL min^{-1})	Fixed-bed quartz-glass flow microreactor (i.d. = 6 mm).	0.2	96	93	18

i.d.- internal diameter of the reactor, vol.- volume

Table S5. Structural parameters of fresh and spent catalysts obtained by Au L_3 edge EXAFS curve fitting analysis.

Sample	Bond	Coordination Number	Bond Length, r (Å)	Debye-Waller factor, σ (Å^2)	R (%)[a]
Au foil	Au–Au	11.8(0.5)	2.85(0)	0.0086(0)	10.1
DPC-RuPt-10-Calc	Au–Au	10.6(1.0)	2.84(0)	0.0085(0)	10.4
DPC-RuPt-10-spent (with air)	Au–Au	10.8(1.0)	2.84(0)	0.0086(0)	10.9
DPC-RuPt-10-spent (without air)	Au–Au	10.9(1.0)	2.84(0)	0.0085(0)	10.8

$^{a}R = \left(\sum(\mathrm{k}^3\chi^{data}(\mathrm{k}) - \mathrm{k}^3\chi^{fit}(\mathrm{k}))^2\right)^{\frac{1}{2}}/\left(\sum(\mathrm{k}^3\chi^{data}(\mathrm{k}))^2\right)^{1/2}$ Figures in parentheses show errors.

Table S6. Stability and corresponding adsorption energy of H atom at various adsorption sites

Site	Stability	E_{dH} (eV)
Ru Surface		
Ru	Stable	-2.56
FCC	Stable	-2.98
HCP	Stable	-3.02
RuPt Surface		
Pt	Stable	-2.86
Pt_2	Stable	-2.70
FCC	Shifts to FCC-bridge	-2.86
HCP	Shifts to HCP-bridge	-3.12

Supplementary References

1. Christopher, P., Xin, H., Marimuthu, A., Linic, S. Singular characteristics and unique chemical bond activation mechanisms of photocatalytic reactions on plasmonic nanostructures. *Nat. Mater*. **11**, 1044–1050 (2012).
2. Yu, S., Jain, P. K. Plasmonic photosynthesis of C1–C3 hydrocarbons from carbon dioxide assisted by an ionic liquid. *Nat. Commun.* **10**, 2022 (2019).
3. Jones G. W. ; Miller, W. E. Ignition temperatures of acetylene-air and acetylene-oxygen mixtures. Report of Investigations, Bureau of Mines, May 1941.

4. Zhou, H., Yang, X., Li, L., Liu, X., Huang, Y., Pan, X., Wang, A., Li, J., Zhang, T. PdZn Intermetallic Nanostructure with Pd–Zn–Pd Ensembles for Highly Active and Chemoselective Semi-Hydrogenation of Acetylene. *ACS Catal.* **6**, 1054–1061 (2016).

5. Feng, Q., Zhao, S., Wang, Y., Dong, J., Chen, W., He, D., Wang, D., Yang, J. Zhu, Y., Zhu, H., Gu, L., Li, Z., Liu, Y., Yu, R., Li, J., Li, Y. Isolated Single-Atom Pd Sites in Intermetallic Nanostructures: High Catalytic Selectivity for Semihydrogenation of Alkynes. *J. Am. Chem. Soc*. **139**, 7294−7301 (2017).

6. Ding, K., Cullen D. A., Zhang, L., Cao, Z., Roy, A. D., Ivanov, I. N., Cao, D. A General Synthesis Approach for Supported Bimetallic Nanoparticles via Surface Inorganometallic Chemistry. *Science* **362**, 560–564 (2018).

7. Guo, Y., Huang, Y., Zeng, B., Han, B., Akri, M., Shi, M., Zhao, Y., Li, Q., Su, Y., Li, L., Jiang, Q., Cui, Y. T., Li, L., Li, R., Qiao, B., Zhang, T. Photo-Thermo Semi-Hydrogenation of Acetylene on Pd_1/TiO_2 Single-Atom Catalyst. *Nat. Commun.* **13**, 2648 (2022).

8. Zou, S., Lou, B., Yang, K., Yuan, W., Zhu, C., Zhu, Y., Du, Y., Lu, L., Liu, J., Huang, W., Yang, B., Gong, Z., Cui, Y., Wang, Y., Ma, L., Ma, J., Jiang, Z., Xiao, L., Fan, J. Grafting Nanometer Metal/Oxide Interface Towards Enhanced Low-Temperature Acetylene Semi-Hydrogenation. *Nat. Commun.* **12**, 5770 (2021).

9. Zhou, S., Shang, L., Zhao, Y., Shi, R., Waterhouse, G. I. N., Huang, Y. C., Zheng, L., Zhang, T. Pd Single-Atom Catalysts on Nitrogen-Doped Graphene for the Highly Selective Photothermal Hydrogenation of Acetylene to Ethylene. *Adv. Mater.* **31**, 1900509 (2019).

10. Huang, F., Deng, Y., Chen, Y., Cai, X., Peng, M., Jia, Z., Ren, P., Xiao, D., Wen, X., Wang, N., Liu, H., Ma, D. Atomically Dispersed Pd on Nanodiamond/Graphene Hybrid for Selective Hydrogenation of Acetylene. *J. Am. Chem. Soc.* **140**, 13142–13146 (2018).

11. Pei, G. X., Liu, X. Y., Yang, X., Zhang, L., Wang, A., Li, L., Wang, H., Wang, X., Zhang, T. Performance of Cu-Alloyed Pd Single-Atom Catalyst for Semihydrogenation of Acetylene under Simulated Front-End Conditions. *ACS Catal.* **7**, 1491−1500 (2017).

12. Armbrüster, M., Kovnir, K., Friedrich, M., Teschner, D., Wowsnick, G., Hahne, M., Gille, P., Szentmiklósi, L., Feuerbacher, M., Heggen, M., Girgsdies, F., Rosenthal, D., Schlögl, R. and Grin, Y. $Al_{13}Fe_4$ as a Low-Cost Alternative for Palladium in Heterogeneous Hydrogenation. *Nat. Mater.* **11**, 690–693 (2012).

13. Hu, M. Z., Zhao, S., Liu, S. J., Chen, C., Chen, W. X., Zhu, W., Liang, C., Cheong, W.-C., Wang, Y., Yu, Y., Peng, Q., Zhou, K. B., Li, J., Li, Y. D. MOF-Confined Sub-2 nm Atomically Ordered Intermetallic PdZn Nanoparticles as High-Performance Catalysts for Selective Hydrogenation of Acetylene. *Adv. Mater*. **30**, 1801878 (2018).

14. Chai, Y., Wu, G., Liu, X., Ren, Y., Dai, W., Wang, C., Xie, Z., Guan, N., Li, L. Acetylene-Selective Hydrogenation Catalyzed by Cationic Nickel Confined in Zeolite. *J. Am. Chem. Soc.* **141**, 9920–9927 (2019).

15. Armbrüster, M., Wowsnick, G., Friedrich, M., Heggen, M., Cardoso-Gil, R. Synthesis and Catalytic Properties of Nanoparticulate Intermetallic Ga–Pd Compounds. *J. Am. Chem. Soc.* **133**, 9112–9118 (2011).

16. Ding, L., Yi, H., Zhang, W., You, R., Cao, T., Yang, J., Lu, J., Huang, W. Activating Edge Sites on Pd Catalysts for Selective Hydrogenation of Acetylene via Selective Ga_2O_3 Decoration. *ACS Catal*. **6**, 3700–3707 (2016).

17. Fu, B., McCue, A. J., Liu, Y., Weng, S., Song, Y., He, Y., Feng, J., Li, D. Highly Selective and Stable Isolated Non-Noble Metal Atom Catalysts for Selective Hydrogenation of Acetylene. *ACS Catal.* **12**, 607−615 (2022).

18. Wei, S., Li, A., Liu, J. C., Li, Z., Chen, W., Gong, Y., Zhang, Q., Cheon, W. C., Wang, Y., Zheng, L., Xiao, H., Chen, C., Wang, D., Peng, Q., Gu, L., Han, X., Li, J., Li, Y. Direct Observation of Noble Metal Nanoparticles Transforming to Thermally Stable Single Atoms. *Nat. Nanotechnol.* **13**, 856–861 (2018).
