# Supplementary material for: Pt-doped Ru nanoparticles loaded on ‘black gold’ plasmonic nanoreactors as air stable reduction catalysts
Source: Nat Commun. 2024 Jan 24;15:713. doi: 10.1038/s41467-024-44954-4 (PMC10808126; doi:10.1038/s41467-024-44954-4)
Supplement: Supplementary file 3 — Description of Additional Supplementary Files [file 41467_2024_44954_MOESM3_ESM.pdf]

Description of Additional Supplementary Files

File Name: Supplementary Data 1
Description: Activation Energy Calculation

File Name: Supplementary Data 2
Description: Quantum Efficiency Calculation Quantum Efficiency Calculation
